# Genetic diversity and structure related to expansion history and habitat isolation: stone marten populating rural–urban habitats

**DOI:** 10.1186/s12898-017-0156-6

**Published:** 2017-12-22

**Authors:** Anna Wereszczuk, Raphaël Leblois, Andrzej Zalewski

**Affiliations:** 1 Mammal Research Institute, Polish Academy of Sciences, Białowieża, Poland; 2 CBGP, INRA, CIRAD, IRD, Montpellier SupAgro, University Montpellier, Montpellier, France; 3 Institut de Biologie Computationnelle, University Montpellier, Montpellier, France

**Keywords:** Genetic structure, Genetic diversity, Isolation, Environmental barrier, Expansion, *Martes foina*

## Abstract

**Background:**

Population genetic diversity and structure are determined by past and current evolutionary processes, among which spatially limited dispersal, genetic drift, and shifts in species distribution boundaries have major effects. In most wildlife species, environmental modifications by humans often lead to contraction of species’ ranges and/or limit their dispersal by acting as environmental barriers. However, in species well adapted to anthropogenic habitat or open landscapes, human induced environmental changes may facilitate dispersal and range expansions. In this study, we analysed whether isolation by distance and deforestation, among other environmental features, promotes or restricts dispersal and expansion in stone marten (*Martes foina*) populations.

**Results:**

We genotyped 298 martens from eight sites at twenty-two microsatellite loci to characterize the genetic variability, population structure and demographic history of stone martens in Poland. At the landscape scale, limited genetic differentiation between sites in a mosaic of urban, rural and forest habitats was mostly influenced by isolation by distance. Statistical clustering and multivariate analyses showed weak genetic structuring with two to four clusters and a high rate of gene flow between them. Stronger genetic differentiation was detected for one stone marten population (NE1) located inside a large forest complex. Genetic differentiation between this site and all others was 20% higher than between other sites separated by similar distances. The genetic uniqueness index of NE1 was also twofold higher than in other sites. Past demographic history analyses showed recent expansion of this species in north-eastern Poland. A decrease in genetic diversity from south to north, and MIGRAINE analyses indicated the direction of expansion of stone marten.

**Conclusions:**

Our results showed that two processes, changes in species distribution boundaries and limited dispersal associated with landscape barriers, affect genetic diversity and structure in stone marten. Analysis of local barriers that reduced dispersal and large scale analyses of genetic structure and demographic history highlight the importance of isolation by distance and forest cover for the past colonization of central Europe by stone marten. This confirmed the hypothesis that human-landscape changes (deforestation) accelerated stone marten expansion, to which climate warming probably has also been contributing over the last few decades.

**Electronic supplementary material:**

The online version of this article (10.1186/s12898-017-0156-6) contains supplementary material, which is available to authorized users.

## Background

The genetic structure of a species is driven by past and ongoing processes within its populations [1–5]. These processes can be divided into two groups: (1) processes within a species range (e.g. affecting population size and densities, and restricting dispersal), and (2) processes caused by changes in the distribution boundaries of a species. Among past processes, the main driver of current genetic structure is the expansion or contraction of a species’ range after changes in biotic or abiotic conditions; species re-colonization of the continent from glacial refuges was the most important of these. Species expansion or contraction is often accelerated by climate changes, and some models predict that even small changes in, for example, average temperature, may result in large shifts in a species range [6, 7]. The second important trigger accelerating expansion or contraction of species range is habitat changes, which can be related to climate changes but also to human-mediated habitat changes (e.g. deforestation and development of large areas of open habitats). In the last 100 years, climate warming and human-induced habitat changes (e.g. land use changes) have driven the northwards expansion of many species, such as the golden jackal [8] and wasp spider [9]. However, in some species expansion or contraction goes unrecorded, as is the case for elusive and shy species for which observations are limited. To fill this knowledge gap, methods other than observation should be used to record changes in range and demographic expansion, and evaluate factors affecting these changes.

A number of genetic signatures may be used to detect demographic changes, particularly range expansions or contractions [10]. Colonization of new areas is due to demographic expansion; thus population growth is one of the most general consequences of range expansion, and it affects genetic diversity and structure [5, 10]. The genetic diversity of an expanded population is related to the process of expansion, which may occur gradually or with serial founder events, as well as to the number of immigrants. During expansion with serial founder events, genetic diversity is higher in the area of initial expansion and gradually decreases along the expansion axis [10, 11]. Both the strength of the founder events and the level of dispersal at the expansion front shape the reduction of genetic diversity at the edge of the species’ range. However, peripheral populations may also undergo differentiation due to genetic drift: rare alleles that reach higher frequencies due to surfing and mutation are characteristic of population frontiers [10–12]. Therefore, all these genetic parameters may show that the population was (or is) in expansion and show the direction of that expansion.

Within a species range, variations in genetic diversity and its structure are often related to population connectivity by gene flow [13, 14]. Animal dispersal is intrinsically linked to biological movement capabilities but can also be restricted by geographic or environmental barriers [15, 16]. Usually such barriers comprise mountains, rivers and other natural landscape features, as well as habitat fragmentation caused by humans. In most cases, habitat modified by humans divides natural landscapes with highly unfavorable habitat (e.g. conversion of forests into open fields); this, in turn, reduces the dispersal rate of animals as connectivity between areas of optimal habitat is disrupted [17, 18]. As barriers reduce—or in extreme cases, preclude—dispersal, gene flow is restricted, which may lead to isolation of populations. Isolated populations suffer from the effects of genetic drift, which leads to loss of genetic diversity and increased differentiation from neighboring populations [19, 20]. Therefore, highly isolated populations, surrounded by very unfavorable anthropogenic habitats (e.g. urban and rural areas) should show high genetic differentiation from neighboring populations and low genetic diversity [21].

Dispersal restricted by anthropogenic habitat changes depends on species adapting to new human-modified habitats. For some species, human-induced changes may enhance dispersal and connectivity and may also accelerate colonization of new areas [8, 9]. Species that have adapted to anthropogenic habitats since Neolithic times can use such areas as optimal habitat [22–24] but may, in turn, find some natural habitats to be unfavorable. Therefore, for these species, large natural areas such as large forest patches could act as barriers reducing dispersal and colonization of new areas is only possible after deforestation and the emergence of more open landscapes within urban or rural areas [9, 23].

The stone marten (*Martes foina*) is a species that has adapted to anthropogenic habitats. It began to colonize Europe alongside Neolithic human societies and is now widespread across large parts of Europe from Portugal to Eastern Russia [23, 25]. Across its geographic range, the stone marten is a habitat-opportunistic carnivore [26]. It occupies a wide variety of habitat types, from woodlands, rocky areas, steppe and semi-desert, fields, pastures, urban and rural areas. In south-western Europe, the stone marten usually occurs in forests, while in central and north-eastern Europe it uses urbanized areas more often [22, 27–29]. Stone marten also inhabit forest-steppe, steppe zones, semi-desert regions and rocky forests in south-west Asia [30]. All this data shows that stone marten habitat use is dependent on its geographical location and is probably constrained by abiotic conditions. The observed preference for urban and rural areas, and avoidance of large, continuous forest complexes without human settlements at the northern edge of its range, suggests that habitat selection is mediated by climatic conditions [27]. This suggestion is in concordance with the hypothesis that explains the history of stone marten colonization of Europe. Sommer and Benecke [23] proposed that the stone marten was late to colonize northern Europe due to a lack of human settlements and large areas of forest cover. The stone marten uses buildings as well-insulated denning sites during winter to mitigate heat loss, and such behavioral thermoregulation probably allows it to survive in areas with cold climates [27]. Therefore, climate warming and the ongoing development of urban and rural areas at the northern edge of its range should have facilitated dispersal and colonization of new areas in the last decades.

Our knowledge about range expansion, colonization history and possible barriers reducing stone marten dispersal at the northern edge of its range is limited. Thus, in this study we used microsatellite markers to: (1) explore the genetic diversity and structure of the stone marten in Poland, (2) investigate its demographic history, in light of the hypothesis about the recent expansion of stone marten populations in north-eastern Europe and (3) analyze the impact on gene flow of potential environmental barriers. We predicted that: (1) stone marten colonized north-eastern Poland in the last decades when the climate warmed and human-induced habitat changed, (2) there are no barriers for dispersal of habitat-opportunistic stone marten in a mosaic of agriculture and natural habitats, but (3) large primeval forest complexes are a barrier for dispersal between populations of this species, and thus affect its pattern of genetic variability.

## Methods

### Sampling and microsatellite genotyping

Tissue samples of 298 individuals were collected in Poland between 1994 and 2015 from eight sites in five regions: north-east (NE), central-east (CE) south-east (SE), south-west (SW) and north-west (NW) Poland. DNA samples were obtained from carcasses of martens killed by cars and from hunters or trapped individuals. We only trapped individuals in NE1 and NE2 sites, where we acquired permission from private land owners to conduct studies on their properties. All marten capture and handling procedures were approved by the Ministry of Environment and the Local Ethics Committee for Animal Experiments at the University of Białystok (no: DL.gł-756/16/98; DL.gł-6713-21/35088/11/abr; DL.gł-6713-14/18806/11/abr; 2011/9). Tissue samples, a 1 cm^2^ piece of skin or muscle, were placed in ethanol and kept frozen at − 20 °C until DNA extraction. The locations of the samples were accurate to 0.5 km.

We extracted DNA from tissue samples using a DNeasy Blood and Tissue Kit (Qiagen) according to the manufacturer’s instructions. Twenty-two microsatellite loci developed for martens were used to genotype all individuals: Ma8, Lut615, Mlut27, Mp0059, Mf3.2, Mf4.10, Mf3.7, Mf6.5, Mvi57, Mvi072, Ma1, Ma2, Gg454, Mel1, Mer041, Mar43, Mar15, Mf4.17, Mf8.8, Mf8.10, Mar08, Mf1.3 [31–40]. Microsatellites were amplified in seven multiplex reactions prepared using a Multiplex PCR Kit (Qiagen) following the manufacturer’s instructions. Reaction mixtures contained approximately 1 μl of template DNA in a total volume of 5.0 μl. The thermal cycle, performed in a DNA Engine Dyad Peltier Thermal Cycler (Bio-Rad), consisted of an initial denaturation step at 95 °C for 15 min, followed by 30 cycles at 94 °C for 30 s, 54, 57 and 60 °C for different multiplex sets for 1 min 30 s, and 72 °C for 1 min, and then a final extension period of 30 min at 60 °C. The amplified fragments were separated by electrophoresis using an ABI 3130XL Genetic Analyzer (Applied Biosystems) with the internal size standard GS500 LIZ™ (Applied Biosystems) using GeneMarker 1.85.

### Genetic diversity

We tested for deviation from Hardy–Weinberg equilibrium and linkage disequilibrium between pairs of loci within each sample site with GENEPOP 4.4 [41] using default parameter values, and Bonferroni’s correction was applied to multiple comparisons. In order to estimate the presence of null alleles we assessed whether heterozygote deficits may be due to null alleles with MICROCHECKER 2.2.3 [42]. Including closely related individuals can increase genetic structure; therefore, we also analysed pairwise relatedness to identify related individuals using the Queller and Goodnight estimator (QGM) [43] implemented in GENALEX version 6.5 [44] and then removed from the dataset one randomly-selected individual from each related pair, defined as pairs with QGM > 0.7. For each site, the genetic variability of each locus, and the mean for all loci were described using the mean allele number (*A*), mean number of private alleles, observed heterozygosity (*H*_O_), unbiased expected heterozygosity (u*H*_E_) and inbreeding coefficients (*F*_IS_) using FSTAT 2.9.3 [45] and GENALEX. The mean number of alleles per locus is expected to be sensitive to sample size; therefore, we also calculated the allelic richness (*Ar*) according to the smallest sample size (N = 12) using FSTAT.

To test the potential influences of different sampling periods on genetic diversity, we compared *Ar* and *H*_E_ for two periods: 1994–2007 and 2008–2015 for NE1, because only from site NE1 did we obtain a minimum of ten individuals in each period. We tested differences among *Ar* and *H*_E_ using Friedman’s test [46].

### Population genetic structure and recent migration

Genetic structure was explored using individual-based Bayesian clustering analyses with the program STRUCTURE v. 2.3.4 (without spatial information) [47], TESS 2.3.1 (incorporating spatial information) [48, 49] and a discriminant analysis of principal components (DAPC) [50]. First, to estimate the most likely number of genetic clusters (K) in STRUCTURE, no prior information about the location of populations was assumed and an admixture model with uncorrelated alleles was used with a burn-in phase of 1,000,000 iterations, followed by a run phase of 1,000,000 iterations. Posterior probability values for the number of clusters (K), ranging from 1 to 10, were calculated from ten independent runs to establish consistency. The most likely number of clusters was determined based on change of the posterior probability of the model, and its rate of change with respect to K using the Δ*K* statistic [51].

Secondly, TESS was run using a Convolution Gaussian prior for spatial admixture (BYM) model with the spatial interaction parameter (*ψ*) set at 0.6 [48]. This parameter weighs the relative importance given to the geographical distance between sites; therefore to check the influence of this parameter we also considered *ψ* at 0.4 and 0.0. We considered ten replicate runs of 20,000 burn-in iterations followed by 30,000 iterations. The number of clusters was set to range from *K* = 2 to *K* = 10. The preferred *K* was selected by comparing the individual assignment results and the deviance information criterion (DIC) [49]. Mean DIC values were plotted against *K* values, and the most likely value of *K* was selected by visually assessing the point at which DIC first reached the plateau of the DIC curve.

Next, DAPC was used to identify genetic clusters by sequential clustering and model selection. This method provides a description of the genetic structuring using coefficients of alleles in linear combinations that give the largest between-group and smallest within-group variances in these loadings. In contrast to analyses in STRUCTURE and TESS, DAPC cluster detection within the genetic data does not consider any assumptions about HW proportions or linkage equilibrium [52]. The most likely number of genetic clusters associated with the lowest Bayesian Information Criterion values was established using the R package adegenet 2.0.1. [52]. We explored values for the number of clusters between 1 and 30 [52]. In order to avoid overfitting of the discriminant functions due to retaining too many PCs, we performed DAPC retaining the optimal number of PCs based on the calculation of the α-score.

The level of genetic differentiation was estimated by *F*_ST_ [53], and Jost’s *D*_EST_ [54], which corrects the *F*_ST_ dependency for the amount of within-site variation, using FSTAT and GENALEX, respectively (significance was assessed by 28,000 permutations for *F*_ST_ and 9999 permutations for *D*_EST_). Differentiation between sample sites based on *F*_ST_ was represented by a dendrogram using the program MEGA v.6 [55]. Genetic differentiation was also described using the genetic uniqueness index (GUI), calculated as the average of the pairwise *F*_ST_ values observed between a site and all other sites [56]. To detect restriction in gene flow between sites in relation to forest cover, the GUI was correlated with the proportion of forest in the buffer zone (20 km width) around each site. Buffer zones and proportions of forests were calculated using ArcGIS 10.2.1 (Environmental Systems Research Institute, Redlands, California).

We tested the presence of isolation by distance (IBD) across the study area using a Mantel test [57]. The Mantel test was performed between a matrix of pairwise genetic distances between sites (*F*_ST_/(1 − *F*_ST_) and the logarithm of geographical Euclidian distance, measured as a straight-line between the central point of each site, using the Isolation by Distance Web Service (http://ibdws.sdsu.edu/~ibdws/) [58], for all sites and next for all sites after removing NE1. In addition, we calculated IBD between NE1 and all other sites separately to analyse the influence of the large forest complex surrounding NE1 on the genetic differentiation of this site. We calculated a simple regression between the pairwise genetic distances and the logarithm of geographical distance for all sites after removing NE1 and next for NE1 and each of the other sites to compare a regression slope.

Current rates of migration between populations were estimated using a Bayesian MCMC method implemented in BIMR 1.0, which is effective at estimating migration rate when genetic structure is weak [59]. Twenty replicates were performed for each MCMC run of 100,000 iterations before sampling (burning), and 20,000 iterations used for posterior estimation (sample size) with a thinning interval of 100. Each of the 20 replicates started with 20 short pilot runs of 1000 iterations each in which incremental values were tuned by the program in an effort to obtain acceptance rates between 25 and 45%. In the next step, the run with the lowest Bayesian deviance (*D*_assign_) was chosen to extract parameter estimates. Posterior densities were visually inspected, and the mode (point estimate) and 95% highest posterior density interval were computed on those densities.

### Past demographic processes

In order to test for recent population contractions or expansions, we used the program BOTTLENECK v.1.2.02 [60]. We tested for heterozygosity excess or deficiency over all loci at each sample site using Wilcoxon signed rank tests based on 10,000 replications. Three models of microsatellite mutation were considered: the stepwise mutation model (SMM), and the two-phase model (TPM) with the variance for mutation size set to 12 and two different values for the proportion of mutations attributed to the SMM—78 and 95% following the recommendations of [61] and [60], respectively. We also used BOTTLENECK to test for a deficit of rare alleles (mode shift) in the distribution of allele frequencies, which is expected if a recent bottleneck had occurred [62].

Populations demographic history was further explored using MIGRAINE 0.5 (http://kimura.univ-montp2.fr/~rousset/Migraine.htm) under single population models with a single continuous past variation in population size (OnePopVarSize) and two past variations in population size (OnePopFounderFlush) [63, 64]. To infer model parameters, the program uses a class of coalescent-based importance sampling algorithms (IS) [64–67]. All analyses were run using a generalized stepwise mutation model (GSM), which is the most realistic model for microsatellite markers and reduces the risk of false positives in bottleneck testing [61, 63]. First, for each sample we inferred point estimates and 95% confidence intervals for the four parameters of the OnePopVarSize model: pGSM, 2*N*µ, 2*N*_*anc*_µ, and *D*g/2*N,* as well as two extra composite parameters, namely *N*_*ratio*_ and Dg*µ, where µ is the mutation rate per generation per locus, *D*g is the time of the demographic change in generations, *N* = *N*_*cur*_ is the current population size and *N*_*anc*_ is the ancestral population size, expressed as the number of genes. Second, we fixed pGSM, the parameter of the geometric distribution of mutation step size under the GSM, at 0.3 based on the results obtained in the previous analysis and inferred the remaining four parameters of the OnePopFounderFlush model: 2*N*µ, 2*N*_*founder*_µ, 2*N*_*anc*_µ and *D*g/2*N*, as well as four extra parameters: *N*_*anc*_ _*ratio*_, *N*_cur_ *N*_*founder*_ _*ratio*_ and *N*_*founder*_ *N*_*anc ratio*_, where *N*_*founder*_ is the founder population size, and Dg*µ. In the OnePopFounderFlush demographic model, *N*_*founder*_ *N*_*anc ratio*_ = *N*_*founder*_ */N*_*anc*_ allows the quantification and testing of a first discrete change in population size (typically a founder event) while *N*_*cur*_ *N*_*founder ratio*_ = *N*_*cur*_ */Nf*_*ounder*_ equivalently characterizes the second continuous change in population size, typically an expansion following the founder event. Extra parameters were used to better characterize the timing, strength and direction of the demographic events, e.g. *N*_*ratio*_ = *N*_*anc ratio*_ = *N*_*cur*_ */N*_*anc*_ quantifies the strength of the change between current and ancestral population sizes—it is < 1 for a contraction, and > 1 for an expansion. Past changes in population size are thus significant when the *N*_*ratio*_’s value 1 lies outside theirs 95% confidence intervals (CI) [63]. To convert scaled parameters (i.e. 2*N*µ, 2*N*_*anc*_µ, 2*N*_*founder*_µ, *D*g/2*N* and Dg*µ) into biological ones (i.e. *N*, *N*_*anc*_, *N*_*founder*_ and *Dg*), we used a mutation rate of 5 × 10^−4^ per locus per generation for all microsatellite loci, a classical average value derived from many different species [68]. We first inferred single past changes in population sizes under the OnePopVarSize demographic model for each of the eight sites separately. Then, we performed the analysis under the OnePopFounderFlush demographic model for sites in which we (1) detected past contractions, and (2) suspected potential past founder events followed by expansions. Analyses under OnePopFounderFlush were thus run separately for the isolated site NE1 and for the pooled sites NE2 + NE3 and CE1 + CE2 due to the lack of genetic structure between them and to increase the signal strength with larger sample sizes. For both demographic models preliminary runs for every dataset were done using 200 points, 200 trees, and 10 iterations. Next, for the final runs, we used 400 points, 50,000 trees, and 10 iterations with narrow parameter ranges deduced from the preliminary runs.

## Results

### Genetic diversity

Twenty-two loci were genotyped for the 298 individuals sampled from the eight study sites. Thirteen individuals with high relatedness to others (QGM > 0.7) and thirteen with missing genotypes were removed from this final dataset. Finally, 272 full multi-locus genotypes were obtained with 0.30% missing data. After sequential Bonferroni correction (p < 0.000027), deviation from HWE was detected in sites NE2 and CE2 for locus Ma1. Null alleles were found for one locus (Ma1) in four sites and for four loci in one or two sites only (Additional file 1: Table S1). Due to the presence of null alleles in 4 sites, locus Ma1 was subsequently excluded from further analysis. Two out of 1848 pairwise locus exact tests of linkage disequilibrium were significant after Bonferroni correction. After excluding Ma1, deviation from HWE was detected in site CE2 only (Table 1). There was no evidence that different sampling periods (1994–2007 vs 2008–2015) had different levels of genetic diversity: allelic richness and expected heterozygosity did not vary between sampling periods (Friedman test; p = 0.225 and p = 0.074, respectively). Therefore we pooled samples from different periods in subsequent analysis.

**Table 1:** Genetic diversity indices of samples of stone marten from eight sites in Poland

Region	Site	N	*A*	*Ar*	Rare *A*	*A* private	*H*_O_	*H*_E_/u*H*_E_	Overall *F*_IS_	HWE (p value)
Northeast	NE1	58	3.57	2.98	22	0.00	0.47	0.47/0.47	− 0.003	0.4095
	NE2	95	4.33	3.40	31	0.33	0.50	0.52/0.52	0.049	0.0001
	NE3	25	3.86	3.45	19	0.14	0.53	0.53/0.54	0.010	0.1117
Central-east	CE1	15	3.81	3.68	20	0.09	0.58	0.54/0.56	− 0.045	0.9519
	CE2	22	3.86	3.50	23	0.05	0.46	0.53/0.54	0.157	0.0000
Southeast	SE1	14	3.90	3.80	15	0.09	0.54	0.57/0.59	0.083	0.0519
Southwest	SW1	31	4.14	3.60	26	0.14	0.52	0.54/0.55	0.055	0.0393
Central-west	NW1	12	3.43	3.43	10	0.00	0.50	0.50/0.53	0.032	0.1023

N, number of samples; *A*, mean number of alleles per locus; *Ar*, allelic richness estimated by rarefaction based on a minimum sample size n = 12; Rare *A*, number of alleles with frequency ≤ 0.07 across all loci; *A* private, private alleles; *H*_O_, observed heterozygosity; u*H*_E_, unbiased expected heterozygosity. The p value cutoff after Bonferroni correction is 0.000027

**Fig. 1:**
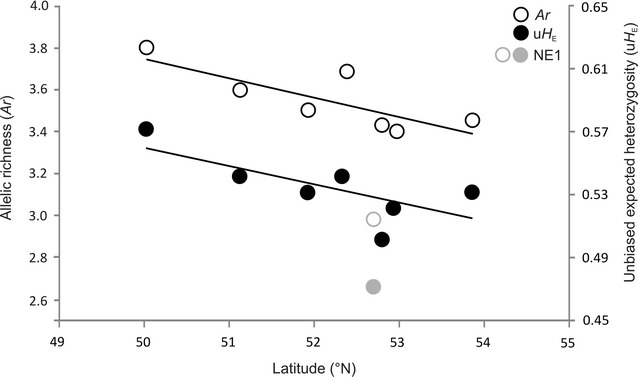
Variation of allelic richness (*Ar*) and unbiased expected heterozygosity (u*H*_E_) of stone marten from eight sites from Poland in relation to latitude. *Ar* and u*H*_E_ of site NE1 were marked separately in grey

The remaining 21 microsatellite loci were polymorphic in all sample sites with a total number of alleles per locus ranging from 3 to 10 and a mean number of alleles per locus of 5.52 (SE ± 0.31). The mean number of alleles (*A*) per locus within each sample site ranged from 3.43 to 4.33, the allelic richness (*Ar*) from 2.98 to 3.80 and the number of private alleles from zero to 0.33 (Table 1). Mean observed heterozygosity (*H*_O_) over all loci was 0.51 (SE = 0.02) and ranged from 0.47 to 0.57, while unbiased expected heterozygosity (u*H*_E_) was 0.53 (SE = 0.02) and ranged from 0.47 to 0.59 (Table 1). Both the number of alleles and allelic richness were lowest in site NE1 (p < 0.0001; Friedman test). After excluding site NE1, allelic richness significantly decreased (Spearman rang correlation, r_S_ = − 0.79, p = 0.048), and expected heterozygosity showed a decreasing trend from south to north (r_S_ = − 0.72, p = 0.067; Fig. 1).

### Patterns of genetic structure, differentiation and isolation by distance

**Fig. 2:**
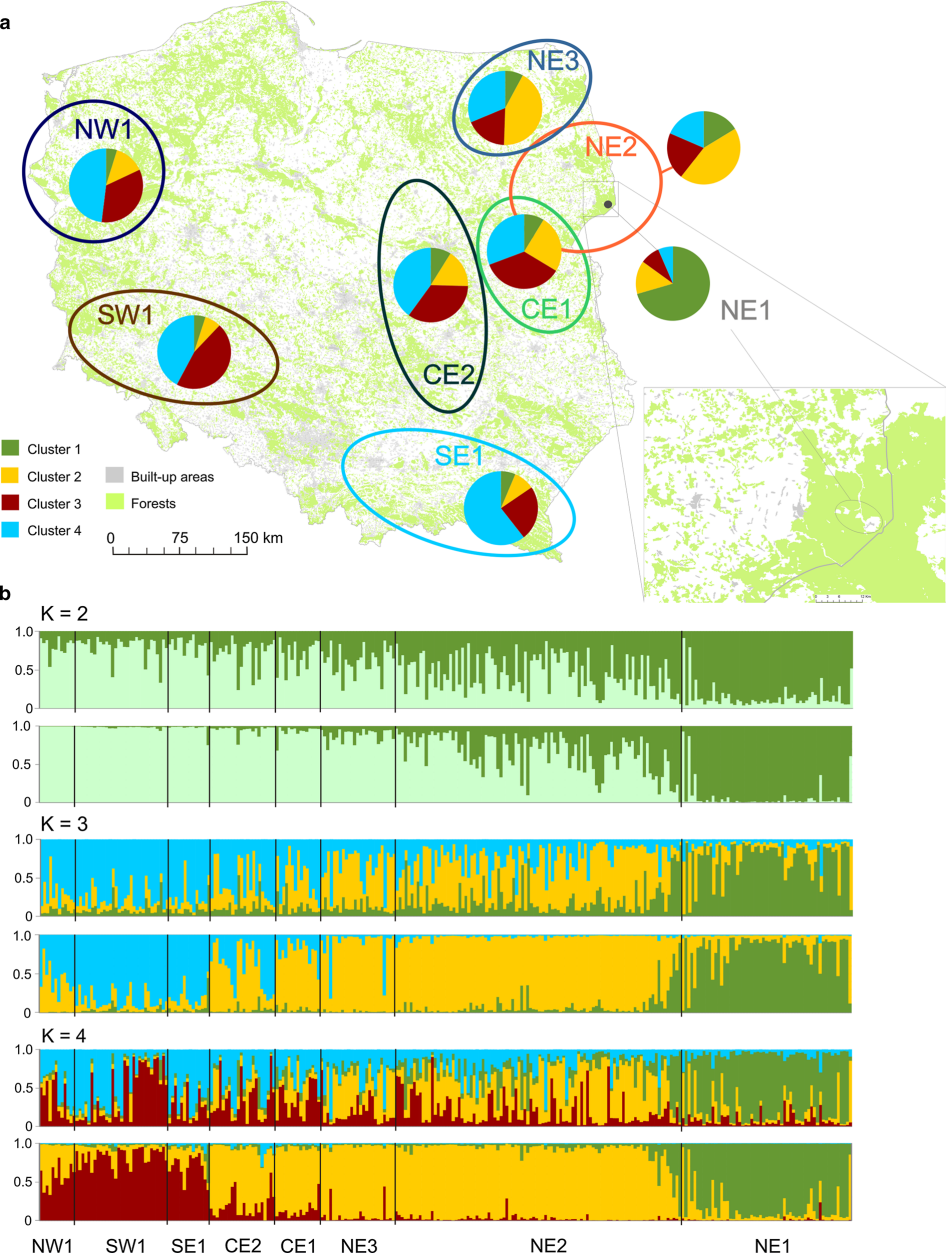
The average proportion of membership identified by STRUCTURE (**a**) and the assignment of stone marten from Poland in the genetic clusters from 2 to 4 (**b**) identified by STRUCTURE (above panel for each K) and TESS (below panel for each K). Single vertical bar represents the individual’s estimated proportion of membership to the genetic cluster. The locality of origin for each individual is indicated below

Cluster analysis with the non-spatial algorithm implemented in STRUCTURE indicated the presence of two to four genetic groups at the uppermost level, supported by the highest maximal posterior probability, the lowest variance between every run and Δ*K* value (Additional file 1: Figure S1). The proportion of martens assigned to cluster 2 (yellow) and 4 (blue) in model K = 4 gradually changed from north-east to south-west. The same pattern of gradually changing proportions of clusters from north-east to south-west was also observed in models K = 2 and K = 3, which is probably due to isolation by distance (Fig. 2). The majority of the sampling areas showed a weak structure, except NE1 in which the proportion of membership was above 70% in all three models K = 2–4 (Fig. 2; Additional file 1: Table S2). Individuals from SW1 also had high membership coefficients (70–80%) in models K = 2–3. TESS gave results similar to those obtained with STRUCTURE. The DIC plot of the TESS runs showed the plateau at K = 3, and additionally the individual assignment was high for only three clusters also at K = 4 (Fig. 2; Additional file 1: Figure S2). Individuals from site NE1 were assigned to cluster 1, cluster 2 was composed of individuals sampled in north-eastern and central Poland (NE2, NE3, CE1, CE2) and cluster 3 was formed by individuals from SE1, SW1 and NW1. TESS produced stable population clusters that were similar and did not change under the influence of different spatial interaction parameters (*ψ*) values (Additional file 1: Figure S3).

**Fig. 3:**
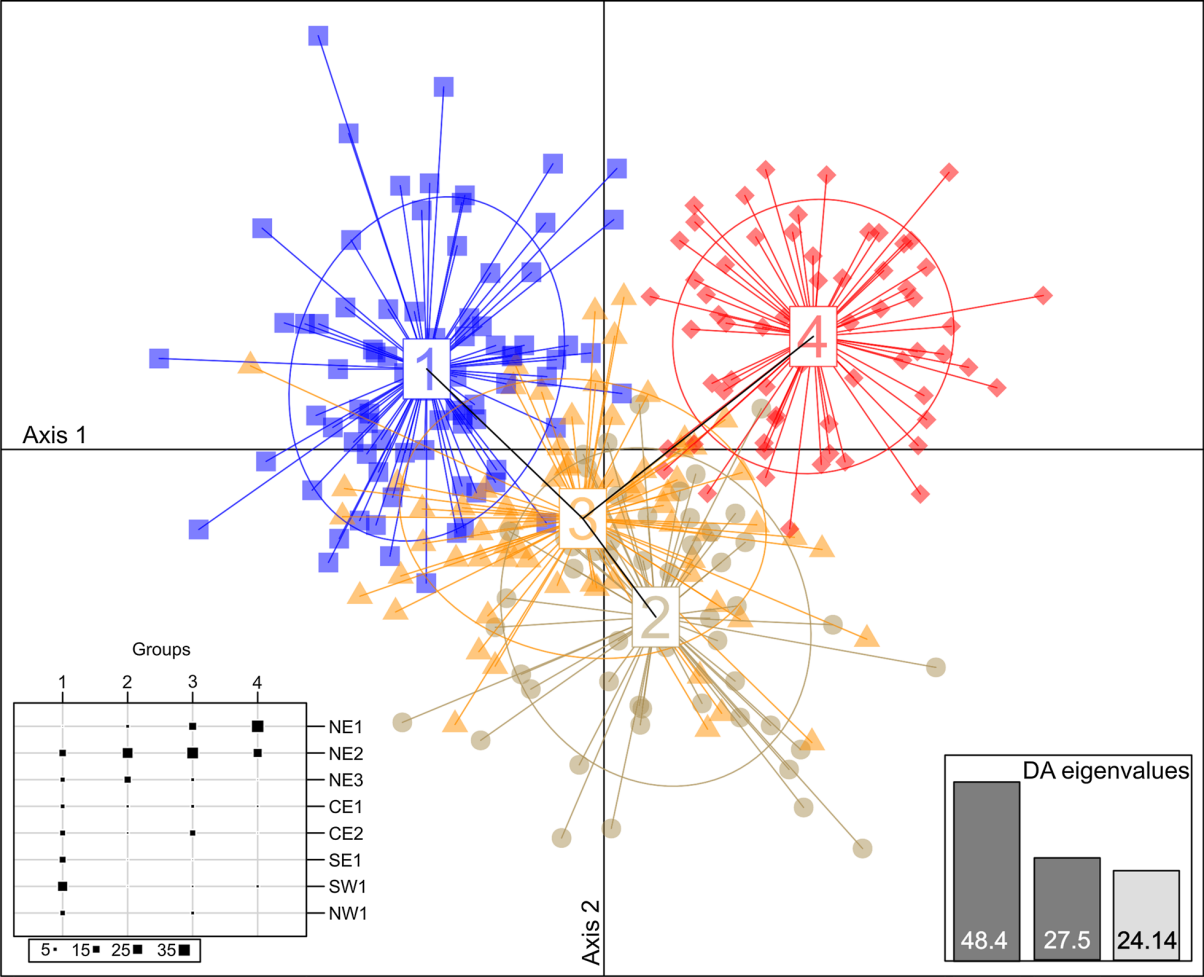
Discriminant analysis of principal components of stone marten from Poland grouped into 4 clusters on the first two axes of DAPC. The main graph plots show the first two discriminant axes (explaining 48.4 and 27.5% of the variation, respectively). Clusters are shown by different colours and shapes, while points represent individuals

The multivariate DAPC identified four clusters based on the Bayesian Information Criterion but they overlap to a large extent (Fig. 3; Additional file 1: Table S2). As for STRUCTURE, the different clusters inferred do not globally correspond to the sampling sites, and each of the four groups consist of individuals from at least two or three sites (Additional file 1: Table S2). Some samples from all sites (except NE1) were assigned to cluster 1, with a higher assignment of samples from SE1, SW1 and NW1 (Fig. 3; Additional file 1: Table S2). The samples from sites NE2, NE3 and CE1 form cluster 2 and samples from NE2, NE3, CE1 and CE2 form cluster 3. In addition, the scatter plot of clusters showed considerable overlap between cluster 3 and clusters 1 and 2 (Fig. 3). Most NE1 samples were separated from all other sites in one cluster (cluster 4) with an admixture of samples from NE2 and no overlap with all other clusters.

**Fig. 4:**
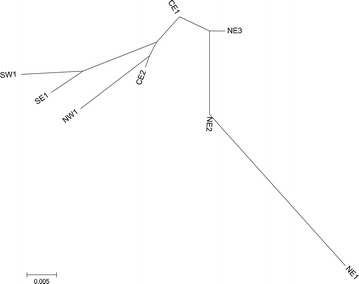
Neighbour-joining tree based on *F*_ST_ values illustrating relationships between populations of stone marten from Poland

Pairwise *F*_ST_ values between sites ranged from 0.002 to 0.084 (Table 2), which indicates relatively limited genetic differentiation patterns between all samples sites. However, NE1 appears more differentiated than the other populations as (1) the largest *F*_ST_ values (from 0.03 to 0.08) were all obtained by comparing NE1 with the other populations; and (2) despite the fact that NE1 is located inside NE2, the *F*_ST_ value between NE1 and NE2 was higher than between NE2 and NE3. Similar results were obtained using *D*_EST_ estimator (Table 2). The largest differentiation was between NE1 and SE1, SW1, NW1, while the lowest was between CE1 and NE2, as well as between CE1 and NE3. The arrangement of branches of the Neighbour-joining tree (Fig. 4), reflecting past divergence events, indicated that SW1, SE1, NW1 and CE2 were the most genetically similar sites, creating one group, whereas sites from NE Poland constituted a separate group. NE1 is the most genetically distant site from all the others and is most genetically similar to NE2.

**Table 2:** Pairwise *F*_ST_ (below diagonal) and Jost’s *D*_EST_ (above diagonal) between samples taken from eight sites in Poland

	NE1	NE2	NE3	CE1	CE2	SE1	SW1	NW1
NE1	–	0.029	0.052	0.051	0.065	0.094	0.084	0.087
NE2	0.0281***	–	0.013	0.002	0.022	0.051	0.058	0.046
NE3	0.0504***	0.0112**	–	0.007	0.024	0.032	0.054	0.034
CE1	0.0493***	0.0016*	0.0055	–	0.005	0.024	0.036	0.022
CE2	0.0624***	0.0192***	0.0204	0.0032	–	0.030	0.041	0.021
SE1	0.0835***	0.0416***	0.0248**	0.0176	0.0225*	–	0.022	0.063
SW1	0.0762***	0.048***	0.0424***	0.0279***	0.033**	0.0169*	–	0.036
NW1	0.0843***	0.0398***	0.0292**	0.0186**	0.0167	0.0467**	0.029*	–
GUI	0.062	0.027	0.026	0.018	0.025	0.036	0.039	0.038

Statistical significance for pairwise *F*_ST_ is given using the adjusted nominal level for multiple comparisons (after Bonferroni correction). Statistically significant values of *F*_ST_ are marked in italics

*GUI* genetic uniqueness index of each site

**Fig. 5:**
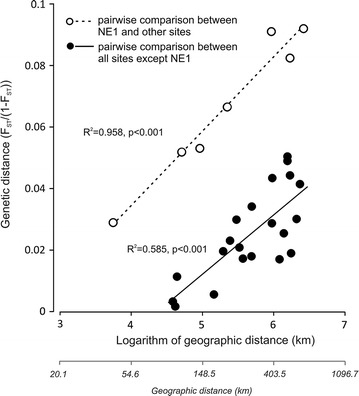
Relationship between stone marten genetic and geographic distance between all sites except NE1 (filled circles) and between NE1 site with 7 other sites (open circles). The regression equation for all sites without NE1 is GenD = 0.019390*GeoD − 0.084829 and for site NE1 vs other 7 sites is GenD = 0.024286*GeoD − 0.063133

The genetic distance was not related to geographic distance for all pairwise comparisons (R^2^ = 0.163, p = 0.059, slope = 0.015), but when NE1 was removed from the analyses the IBD relationship significantly explained a significant proportion of the variation (R^2^ = 0.585, p < 0.001, slope = 0.019). The slope of regression of genetic and geographic distances from NE1 to other sites was similar to slope between distance matrices excluding NE1 (R^2^ = 0.958, p < 0.001, slope = 0.024). The mean differentiation between NE1 and the other sites was on average 20.2% higher than the regression line of genetic vs geographic distances of martens populations between the other sites (i.e. similar slope but much larger intercept, Fig. 5). The genetic uniqueness index increased with proportion of forests in the 20 km-buffer around each site (Spearman rank correlation, r_S_ = 0.78, p = 0.028; Additional file 1: Figure S4) and was highest for site NE1 (Table 2).

### Recent migration and past demographic processes

Results of BIMR analysis of the migration rate showed no migration between most sites. Asymmetric gene flow into the NE2 population from the rest of the populations over the last generation was detected, with no migration from NE2 to the other sites (Additional file 1: Table S3). Migration between NE1 and NE2 sites appears restricted and asymmetrical: there was some migration from NE1 to NE2 but no migration from NE2 to NE1, despite the fact that both sites are close to each other. We found no evidence of immigration or emigration between any other pairs of sites (Additional file 1: Table S3).

Past demographic changes were first analyzed using BOTTLENECK and revealed no significant reduction in population sizes under a SMM, while under a TPM:0.22 a marginally significant heterozygosity excess, indicating past contraction, was detected in site SW1 (Additional file 1: Table S4). None of the eight sites showed evidence of a heterozygosity deficiency indicating expansion. The mode shift test revealed a normal L-shaped distribution indicating the lack of recent loss of rare alleles that is often found in populations that have undergone recent severe bottleneck, with the exception of NE3 and CE1 (Additional file 1: Table S4).

**Fig. 6:**
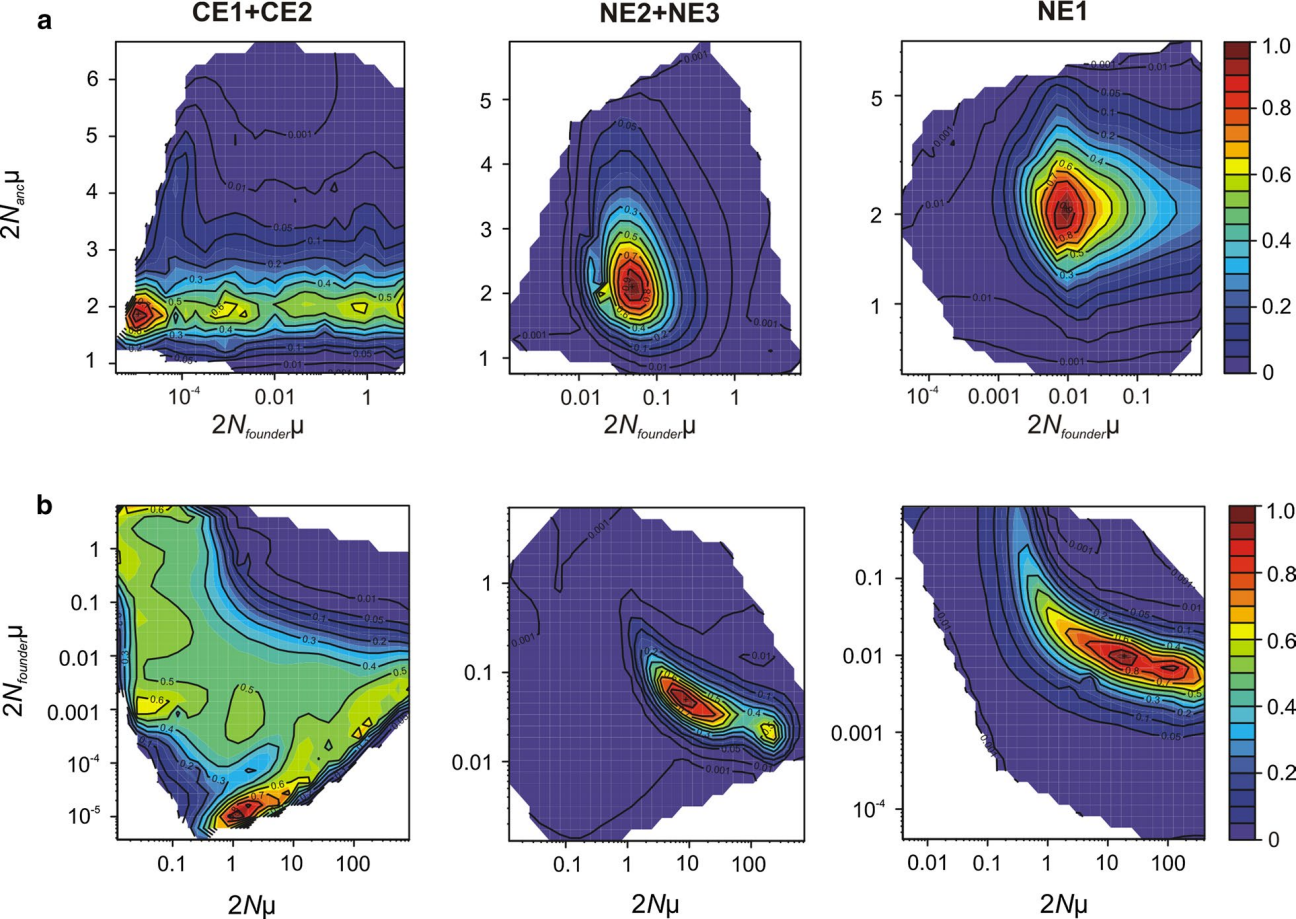
Pairwise likelihood-ratio profiles obtained with MIGRAINE under the OnePopFounderFlush model for central sites (pooled CE1 and CE2), north-eastern sites (pooled NE2 and NE3) and site NE1 from Poland for some pairs of the following parameters: 2 *N*_*anc*_µ: ancestral effective population size; 2*N*_*founder*_µ: founder population size; 2*N*µ: current effective population size. Very recent founder events (panel **a**) were detected for NE2 + NE3 and NE1 and significant expansion (panel **b**) for NE2 + NE3, contrasting with a stable population for sites CE1 + CE2. All axes are represented using a log scale. Point estimate values for each parameter and the associated 95% confidence interval are shown in Additional file 1: Table S6

In contrast to BOTTLENECK, the analyses of past demographic history with the GSM assumption and OnePopVarSize demographic model using MIGRAINE indicated significant contractions in sites NE1, NE2 and pooled CE1 + CE2 and a nearly significant contraction in site NE3 (Additional file 1: Table S5). Populations from south-west Poland remained stable and reach the highest numbers of individuals, which gradually decrease towards the north-east. Inferred population size for NE1 was 160 individuals; however, there is not enough information on the strength and timing of past processes, and the bounds of the confidence intervals may be more informative (Additional file 1: Table S5). To get more detailed information on past demographic changes we carried out an analysis under the OnePopFounderFlush model for sites NE1, NE2 + NE3 and CE1 + CE2, which indicated a significant founder event for NE1 (Fig. 6a; Additional file 1: Table S6) followed by a non-significant expansion (Fig. 6b; Additional file 1: Table S6, Figure S5). Results for the pooled sites of north-eastern Poland (NE2 + NE3) showed a significant founder event (Fig. 6a) followed by a significant expansion (Fig. 6b), whereas populations from central Poland (CE1 + CE2) demonstrated signs of a stable population. The scaled time, in generations, of the founder events in the populations in which we found them, showed a very recent founder event in NE1 (17 generations ago; CI 4–336) and a relatively recent founder event in NE2 + NE3 (45 generations ago; CI 18–142).

## Discussion

In this study we demonstrate the influence of processes acting within a range and shaping species’ range boundaries on population genetic structure and diversity. Moderate genetic diversity and low genetic structure of stone marten at the landscape scale were related to recent expansion to north-eastern Poland and a general lack of environmental barriers limiting dispersal of this species. The genetic structure was mainly related to isolation by distance, with a gradual cline in genetic differentiation over increasing geographic distance. However, the data also showed strong evidence for the isolation of one sampled population vs the others, probably due to an environmental barrier—a large forest complex. Isolation, restriction of migration and genetic drift were the primary factors that resulted in reduced genetic diversity in this population.

### Isolation by barrier

Our results showed that a large forest complex may be a major impediment to dispersal and gene flow between stone marten populations. This was confirmed by various analyses comparing a site surrounded by a large forest complex (NE1) with other sites, including a neighboring site 20 km away. First, pairwise comparisons between NE1 and other sites gave the highest *F*_ST_ values, which were significantly greater than zero. Second, the analyses of the Neighbor-joining tree showed that NE1 is the most distantly related to the other sites on the tree. The IBD analysis showed that the genetic distance from NE1 to other sites is 20% higher than between other sites separated by similar geographic distances. Furthermore, Bayesian clustering implemented in STRUCTURE and DAPC, with no a priori information on an individual’s origin, grouped most samples from NE1 into one cluster with a high probability of assignment and with a small admixture with individuals from site NE2. These results are similar to results obtained from isolated populations on islands that are strongly genetically differentiated and form clearly separate clusters [68–71].

The distinct genetic structure of NE1, an isolated population inside a large forest complex, has probably been maintained by low gene flow from neighboring sites. In general, isolated populations with low gene flow are prone to losing alleles, reducing genetic diversity and viability as a result of genetic drift [71–74]. Due to asymmetric migration (BIMR analysis) and rare gene flow into NE1, we may expect higher effect of genetic drift, leading to a reduction in genetic diversity. The genetic diversity of stone marten from NE1 was the lowest among all study sites and lower than in neighboring sites 20 km away. Stone martens inhabiting NE1 exhibited signatures of genetic drift, having lower levels of allelic richness as well as lower expected and observed heterozygosities than populations inhabiting other sites. The low expected (*H*_E_ = 0.47) and observed heterozygosity (*H*_O_ = 0.47) of NE1 are similar to results of isolated populations of other mammals, e.g. the edible dormouse *Glis glis* (*H*_E_ < 0.42), Ethiopian wolf *Canis simensis* (*H*_E_ = 0.38–0.54) and fisher *Pekania pennanti* (*H*_E_ = 0.47–0.56) [74–77]. Despite a great reduction in *Ar* and u*H*_E_, there was no evidence of bottlenecks (analysis using BOTTLENECK). However, analyses with MIGRAINE showed significant founder and expansion events in site NE1, suggesting that stone marten recently colonized this area and that the low local genetic diversity at this site may be related to the founder event.

The large genetic differentiation in and low migration rate into NE1 confirmed that this stone marten population is separated from the other sites by a large forest complex, creating an isolated population. The avoidance of this large forest complex by stone marten in contrast to pine marten (*Martes martes*) has been confirmed by habitat selection analyses of radio-tracked martens [27]. Stone marten possibly avoid large forests to reduce thermal stress in winter, avoid predators and due to their food preferences [27]. The stone marten originally evolved in Central Asia and is probably adapted to a subtropical climate; thus it selects the most insulated resting sites available, preferring human buildings rather than tree cavities in forest. In addition, its less arboreal lifestyle compared to pine marten exposes it to an increase risk of predation in forests, when moving or resting on or under the ground [22]. Furthermore, villages, in contrast to forests, offer a high abundance of food throughout the year [78]. The genetic analyses in this study show, for the first time, that a large forest complex is not only a habitat that is avoided by stone marten but also constitutes an environmental barrier in the dispersal of this species.

Comparison of the genetic diversity and genetic structure of stone marten from all other sites (except site NE1) suggested an absence of barriers and high dispersal rates between sites, causing low genetic structuring across large parts of Poland. However, genetic uniqueness of martens inhabiting sites increased with the increase proportion of forest cover around the sites. This analysis confirmed that forests may restrict gene flow between sites but that probably only large forest complexes reduce it to a level that affects genetic structure. A lack of distinct genetic structure and small divergence between all sites except NE1 was indicated by *F*_ST_ pairwise comparisons between sites and significant isolation by distance patterns. The genetic structure of stone marten in Poland revealed two to four clusters with high levels of admixture among sites and low probability of assignment (except site NE1). This was confirmed by clustering methods implemented in STRUCTURE, TESS and DAPC. Greater structures in stone marten populations, with higher probabilities of assignment to groups have been observed elsewhere in Europe (France, Portugal and Spain); however the geographic areas considered in these studies were slightly larger than in our study [78–81]. A weak genetic structure for the stone marten population in Poland seems to confirm the absence of ecological barrier-restricted dispersal. In central Europe, stone marten mostly inhabit villages and towns but also small patches of forest, especially in fragmented agricultural landscapes [22, 27]. In addition, they prefer shrub, ecotone areas and brushwood during movement and dispersal [22], which are widely available in human-modified landscapes. In central and western Poland, the distribution and density of these habitat patches probably provides sufficient connectivity for undisturbed marten migration. A low migration rate between sites in southern and eastern Poland (BIMR analysis), which lack genetic structure, suggests a “stepping-stone” migration model where study sites are too distant from each other for identification of direct recent migrants.

### Expansion in Poland

The lack of evident genetic structure and large similarity between stone marten inhabiting distantly-located sites in Poland may also reflect demographic processes, notably the history of colonisation of Poland by this species. The signal of expansion detected by MIGRAINE indicates a recent demographic expansion of stone marten in NE Poland. The arrangement of Neighbor-joining tree branches indicated the directions of population expansions: the populations from SW Poland gave rise to the populations of central Poland and individuals from these populations then expanded into the north-eastern regions. Also, the DAPC genetic structure results suggested the direction of differentiation of populations, where group 1—consisting of the south-west sites—originated group 3 (central and north-east sites), which in turn initiated the existence of the most north-eastern group 2 and isolated group 4. The direction of migration, indicated by BIMR analysis, from all sites to NE2 may also reflect the direction of current expansion. Furthermore, genetic diversity usually decreased along the expansion axis [2, 82]. In Poland *Ar* and u*H*_E_ decreased from south-west to north-east Poland; the highest genetic diversity was recorded in SE1 site and *Ar* or u*H*_E_ decreased to the north, both north-west or north-east. The gradual loss of genetic variability is typical during colonization of new territories because of population bottlenecks and founder effects [2, 82].

**Fig. 7:**
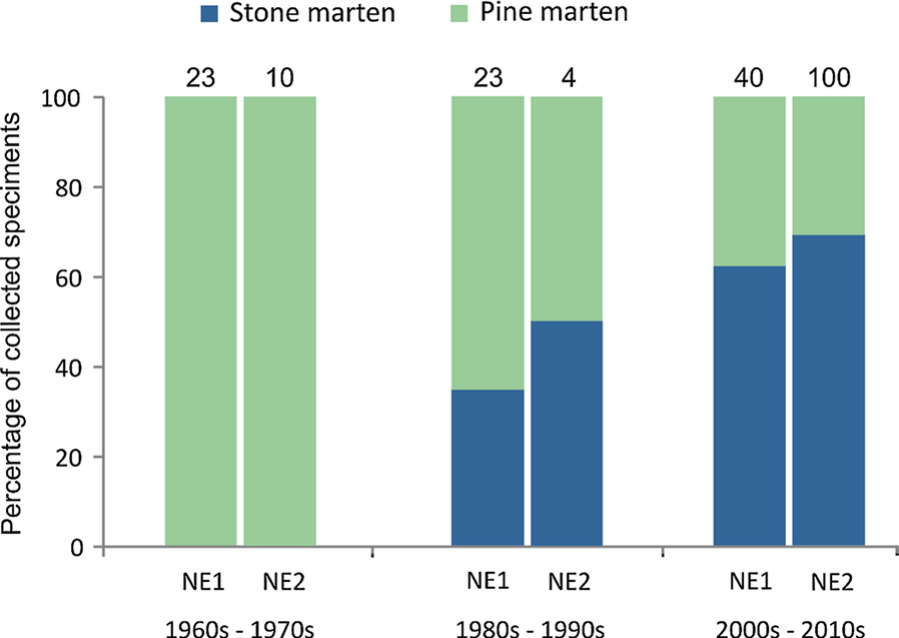
Percentage of collected stone and pine marten from NE1 and NE2 sites in collection of Mammal Research Institute PAS, every 20 years from 1960 to 2010. The numbers above bars indicate sample size

The past demographic inferences obtained with MIGRAINE analyses suggested stone marten colonized NE Poland relatively recently (17–45 generations), which is confirmed by observations of stone marten presence in this region. Assuming a generation time of 2 years we can estimate the time of colonization to be around 34–90 years ago but this estimation should be treated with caution as it may be slightly biased by non-synchronous sampling. The proportion of stone to pine martens collected from hunters and road-killed animals in the zoological collection of the Mammal Research Institute, Polish Academy of Sciences has increased, over successive decades, since the 1980s (Fig. 7). In NE Poland, no stone marten were collected during the 1960s and 1970s; the first stone marten were collected in the ‘80s and its proportion relative to the number of pine marten collected gradually increased over subsequent decades (Fig. 7). Furthermore, in NE Poland only three observations of stone marten were recorded between 1960 and 1974 in the “Atlas of Polish mammals” [83]. During this period the authors did not find any evidence of stone marten presence in church lofts (excrement, eggshells or other prey eaten by stone marten), while successfully having found it in other parts of Poland, where they identified 54 such cases [83]. All of these observations support our findings of likely recent expansion of stone marten in NE Poland or repopulation after a significant decline, which took place before the 1960s. In contrast to central and eastern Poland, which has higher numbers of villages, cities and small fragments of forest, NE Poland still has large forest complexes and the number of villages is lower. A lack of anthropogenic habitat probably slowed down the expansion of stone marten in NE Poland. We suggest however, that climate warming may also be a factor that is now accelerating the expansion. The hypothesis that climate severity caused the stone marten to avoid large forest complexes at the north-east boundary of its range [27] suggests that climate warming over the last two decades [84] may have allowed the stone marten to disperse across smaller forest patches and accelerate its invasion of new areas at the edge of its range.

The stone marten probably colonized Europe post-8000 YBP, which was associated with the spread of Neolithic societies [85, 86]. In the late Mesolithic and early Neolithic, the stone marten was recorded in central Europe, particularly in France and Italy. From France, it arrived in the south of the Iberian Peninsula about 7000 YBP; about 3000–5000 YBP stone marten reached the north of the Iberian Peninsula [23]. The stone marten has been colonizing north-eastern Europe since the thirteenth century, since the development of a denser network of human settlements and larger forest fragmentation. Earlier colonization of this region was probably restricted due to a lack of human settlements [23, 81]. The comparison of genetic variability of stone marten in Poland to southern Europe confirmed this colonization history. Assuming that the stone marten first colonized from south-eastern to south-western Europe and then from south to north, we may expect a gradually increase of number of alleles per locus between these sites. Indeed, comparisons of genetic diversity between our and other studies confirm this: the differences in measures of genetic diversity (mean allele number and *H*_E_) for the same loci (as used in this study) were higher when considering populations from southern Europe (Bulgaria) than when considering populations from south-western Europe (France, Spain and Portugal; Table 3) [80, 81, 87]. The colonization of new areas by the stone marten is still ongoing and its range has increased in northern and eastern Europe over the last two decades [25]. Marten expansion is also ongoing in eastern Europe and Asia; since the 1980s stone marten have colonized areas east of the Volga River [30]. This ongoing expansion may be interpreted as a demographic response to habitat changes and global climate warming and are consistent with our observation of stone marten expansion in NE Poland.

**Table 3:** Comparison of mean allele number and expected heterozygosity for loci shared between this study and the studies from other part of Europe

Country	*N* shared loci	Mean allele number (SE)		Expected heterozygosity		Sources
		Other study	This study	Other study	This study	
Bulgaria	3	6.67 (0.33)	3.67 (0.33)	0.709–0.818	0.230–0.607	[87]
Spain and Portugal	15	7.4 (0.50)	6.0 (0.52)	0.375–0.854	0.219–0.751	[81]
France	6	8.33 (1.04)	7.33 (1.02)	0.406–0.844	0.447–0.751	[80]

## Conclusions

Our results showed that two processes, namely changes in species distribution boundaries and limited dispersal associated with landscape barriers within the species range, probably affect genetic diversity and structure in stone marten in Poland. In landscapes less modified by humans, with large forest complexes, dispersal is limited, and structure between stone marten populations increases. Analysis of local barriers that reduced dispersal and large scale analyses of genetic structure and diversity highlight the importance of anthropogenic landscapes for the past colonization of central Europe by stone marten. This confirmed the hypothesis that human-landscape changes accelerated stone marten expansion, to which climate warming has also probably been contributing over the last few decades. The ongoing colonization process in north-eastern Europe and Asia may be explained by these two changes in biotic and abiotic conditions.

## Additional files

**Additional file 1.** Additional figures and tables.

**Additional file 2.** Row experimental data.

## Abbreviations

NE: north-east region of Poland
CE: central-east region of Poland
SE: south-east region of Poland
SW: south-west region of Poland
NW: north-west region of Poland
*A*: mean allele number
*H*_O_: observed heterozygosity
u*H*_E_: unbiased expected heterozygosity
*F*_IS_: inbreeding coefficients
*Ar*: allelic richness
DAPC: discriminant analysis of principal components
SMM: stepwise mutation model
TPM: two-phase model
GSM: generalized stepwise mutation model
CI: confidence intervals
IBD: isolation by distance
GUI: genetic uniqueness index
