# Supplementary material for: Genetic diversity and structure related to expansion history and habitat isolation: stone marten populating rural–urban habitats
Source: BMC Ecol. 2017 Dec 22;17:46. doi: 10.1186/s12898-017-0156-6 (PMC5741947; doi:10.1186/s12898-017-0156-6)
Supplement: Supplementary file 1 — Additional file 1. Additional figures and tables. [file 12898_2017_156_MOESM1_ESM.docx]

**Additional material to the manuscript**

*Genetic diversity and structure related to expansion history and habitat isolation: stone marten populating rural-urban habitats*

Anna Wereszczuk, Raphaël Leblois and Andrzej Zalewski

**Table S1**. Summary of results afforded using MICROCHECKER (10 000 Monte Carlo simulations) in 8 sites of stone marten (*Martes foina*) from Poland. In parentheses sample size.

| Locus | NE1 | NE2 | NE3 | CE1 | CE2 | SE1 | SW1 | CW1 |
| --- | --- | --- | --- | --- | --- | --- | --- | --- |
|  | (58) | (95) | (25) | (15) | (22) | (14) | (31) | (12) |
| Ma8 | ns | ns | ns | ns | ns | ns | ns | ns |
| Lut615 | ns | ns | ns | ns | ns | ns | ns | ns |
| Lut27 | ns | ns | ns | ns | ns | ns | ns | ns |
| Mp059 | ns | ns | ns | ns | ns | ns | ns | ns |
| Mf3.2 | ns | ns | ns | ns | ns | ns | ns | ns |
| Mf4.10 | ns | ns | ns | ns | ns | ns | ns | ** |
| Mf3.7 | ns | ns | ns | ns | ns | ns | ns | ns |
| Mf6.5 | ns | ns | ns | ns | ns | ns | ns | ns |
| Mvi57 | ns | ns | ns | ns | ns | ns | ns | ns |
| Mvi072 | ns | *** | ns | ns | ns | ns | ns | ns |
| Ma2 | ns | ns | ns | ns | ns | ns | ns | ns |
| Gg454 | ns | ns | ns | ns | ns | ns | ns | ns |
| Mel1 | ns | ns | ns | ns | ns | ns | ns | ns |
| Mer041 | ns | ns | ns | ns | *** | ns | *** | ns |
| Mer43 | ns | ns | ns | ns | ns | ns | ns | ns |
| Mer15 | ns | ns | ns | ns | ns | ns | ns | ns |
| Mf4.17 | ns | ns | ns | ns | * | ns | ns | ns |
| Mf8.8 | ns | ns | ns | ns | ns | ns | ns | ns |
| Mf8.10 | ns | * | * | ns | ns | ns | ns | ns |
| Mer08 | ns | ns | ns | ns | ns | ns | ns | ns |
| Mf1.3 | ns | ns | ns | ns | ns | *** | ns | ns |
| Ma1 | *** | *** | *** | ns | *** | ns | ns | ns |

Level of significance: ns – not significant; * P<0.05; ** p<0.01; *** p<0.001

**Figure S1.** Posterior probabilities (LnP(K)) for (a) each independent run and (b) average for the 10 runs of STRUCTURE on the data set (N=272) and corresponding ΔK for K between 1 and 10.

**(a)**


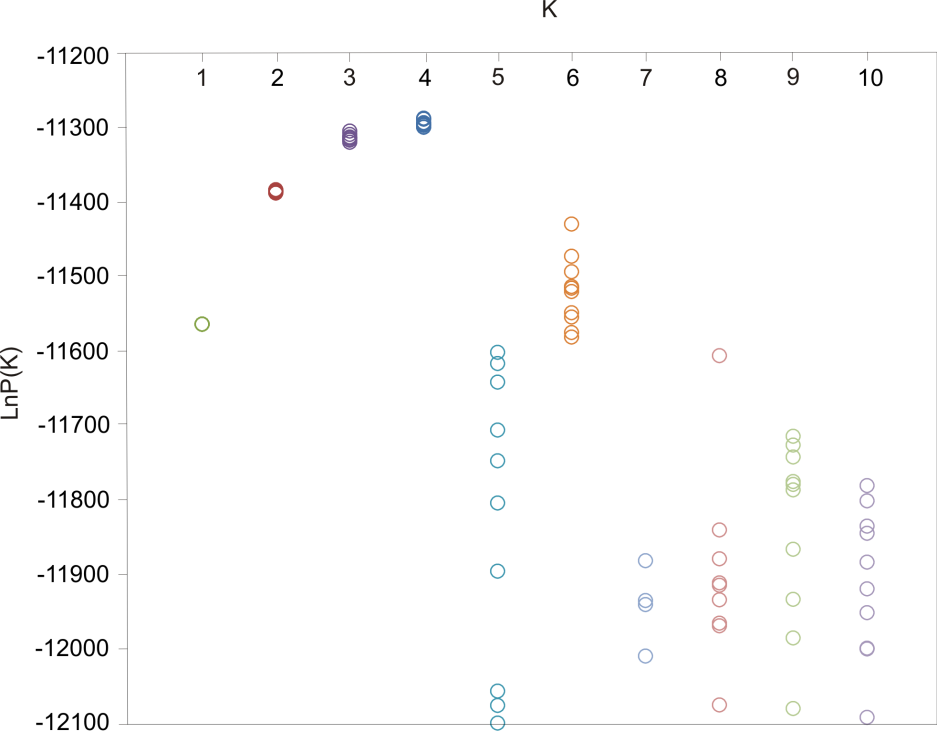


**(b)**

**
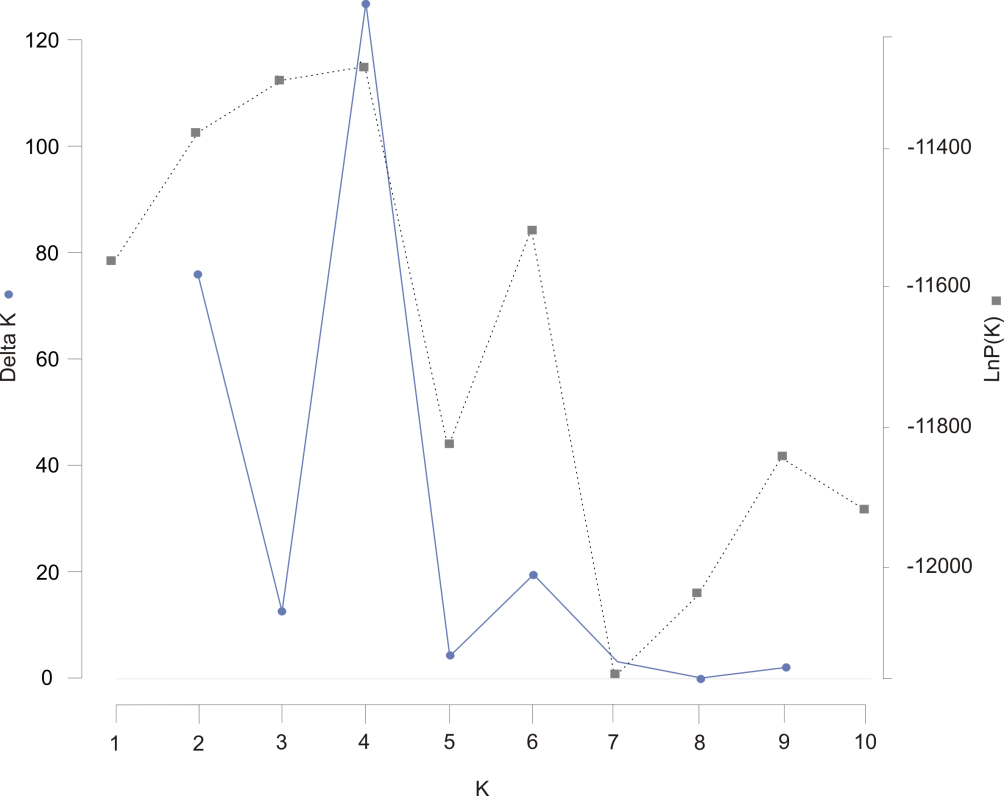
**

**Table S2.** The average proportion of membership for the clusters identified by STRUCTURE and DAPC of stone marten in Poland.

| Region | Site | N | STRUCTURE | | | | | | | | | | |
| --- | --- | --- | --- | --- | --- | --- | --- | --- | --- | --- | --- | --- | --- |
|  |  |  | K=2 | |  | K=3 | | |  | K=4 | | | |
|  |  |  | Cluster 1 | Cluster 2 |  | Cluster 1 | Cluster 2 | Cluster 3 |  | Cluster 1 | Cluster 2 | Cluster 3 | Cluster 4 |
| Northeast Poland | NE1 | 58 | **0.846** | 0.154 |  | **0.744** | 0.176 | 0.080 |  | **0.705** | 0.145 | 0.085 | 0.065 |
|  | NE2 | 95 | 0.531 | 0.469 |  | 0.217 | 0.549 | 0.234 |  | 0.163 | 0.443 | 0.209 | 0.185 |
|  | NE3 | 25 | 0.383 | 0.617 |  | 0.117 | 0.561 | 0.322 |  | 0.079 | 0.426 | 0.182 | 0.313 |
| Central-east Poland | CE1 | 15 | 0.304 | 0.696 |  | 0.128 | 0.369 | 0.503 |  | 0.087 | 0.249 | 0.356 | 0.307 |
|  | CE2 | 22 | 0.277 | **0.723** |  | 0.130 | 0.299 | 0.570 |  | 0.089 | 0.165 | 0.346 | 0.400 |
| Southeast Poland | SE1 | 14 | 0.193 | **0.807** |  | 0.098 | 0.148 | **0.753** |  | 0.064 | 0.091 | 0.239 | 0.606 |
| Southwest Poland | SW1 | 31 | 0.197 | **0.803** |  | 0.099 | 0.146 | **0.755** |  | 0.050 | 0.071 | 0.457 | 0.422 |
| Central-west Poland | NW1 | 12 | 0.190 | **0.810** |  | 0.080 | 0.223 | **0.697** |  | 0.048 | 0.132 | 0.339 | 0.480 |
|  |  |  | DAPC | | | | | | | | | | |
| Northeast Poland | NE1 | 58 | **0.833** | 0.166 |  | 0.648 | 0.032 | 0.036 |  | 0.033 | 0.085 | 0.249 | 0.632 |
|  | NE2 | 95 | 0.510 | 0.490 |  | 0.191 | 0.574 | 0.234 |  | 0.180 | 0.276 | 0.338 | 0.206 |
|  | NE3 | 25 | 0.275 | **0.725** |  | 0.097 | 0.506 | 0.397 |  | 0.261 | 0.435 | 0.242 | 0.062 |
| Central-east Poland | CE1 | 15 | 0.322 | 0.678 |  | 0.072 | 0.510 | 0.418 |  | 0.410 | 0.189 | 0.320 | 0.081 |
|  | CE2 | 22 | 0.167 | **0.833** |  | 0.062 | 0.387 | 0.55 |  | 0.400 | 0.099 | 0.448 | 0.053 |
| Southeast Poland | SE1 | 14 | 0.084 | **0.916** |  | 0.116 | 0.073 | **0.810** |  | **0.783** | 0.084 | 0.082 | 0.051 |
| Southwest Poland | SW1 | 31 | 0.099 | **0.901** |  | 0.099 | 0.079 | **0.822** |  | **0.788** | 0.054 | 0.068 | 0.089 |
| Central-west Poland | NW1 | 12 | 0.030 | **0.97** |  | 0.004 | 0.23 | **0.772** |  | **0.615** | 0.097 | 0.284 | 0.004 |

The proportion of membership above 0.7 is marked in bold. N – sample size.

**Figure S2.** The deviance information criterion (DIC) scores computed by the TESS 2.3.1 admixture model plotted against K, where K = 3 is indicated as the model that best fits the data.

**
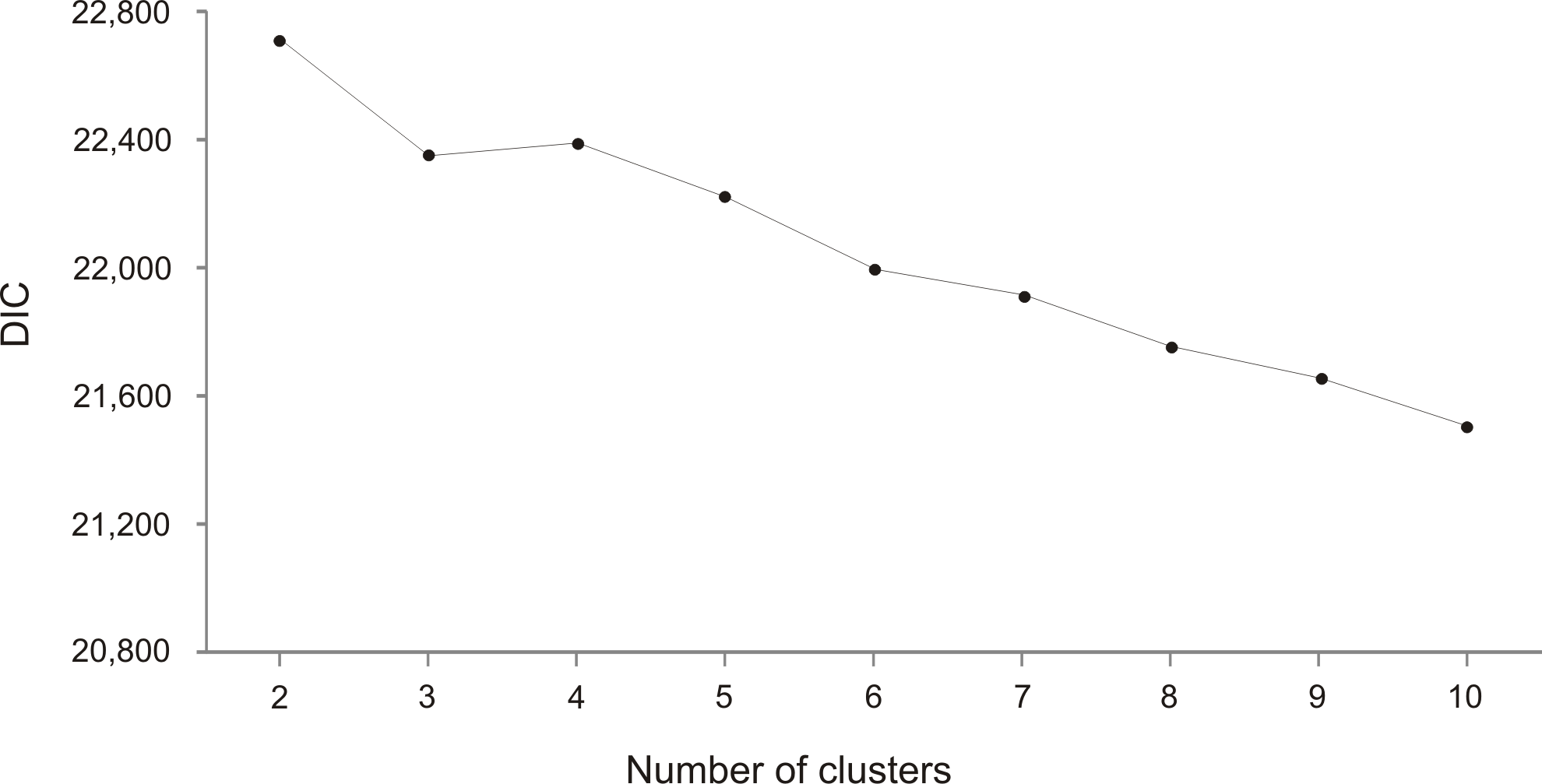
**

**Figure S3.** Individual assignment probabilities of stone martens from eight sites in Poland to genetic clusters using TESS admixture model for K = 2, K = 3 and K = 4 and different spatial interaction parameters. Single vertical bar represents the individual’s estimated proportion of membership to the genetic cluster. The locality of origin for each individual is indicated below.

**
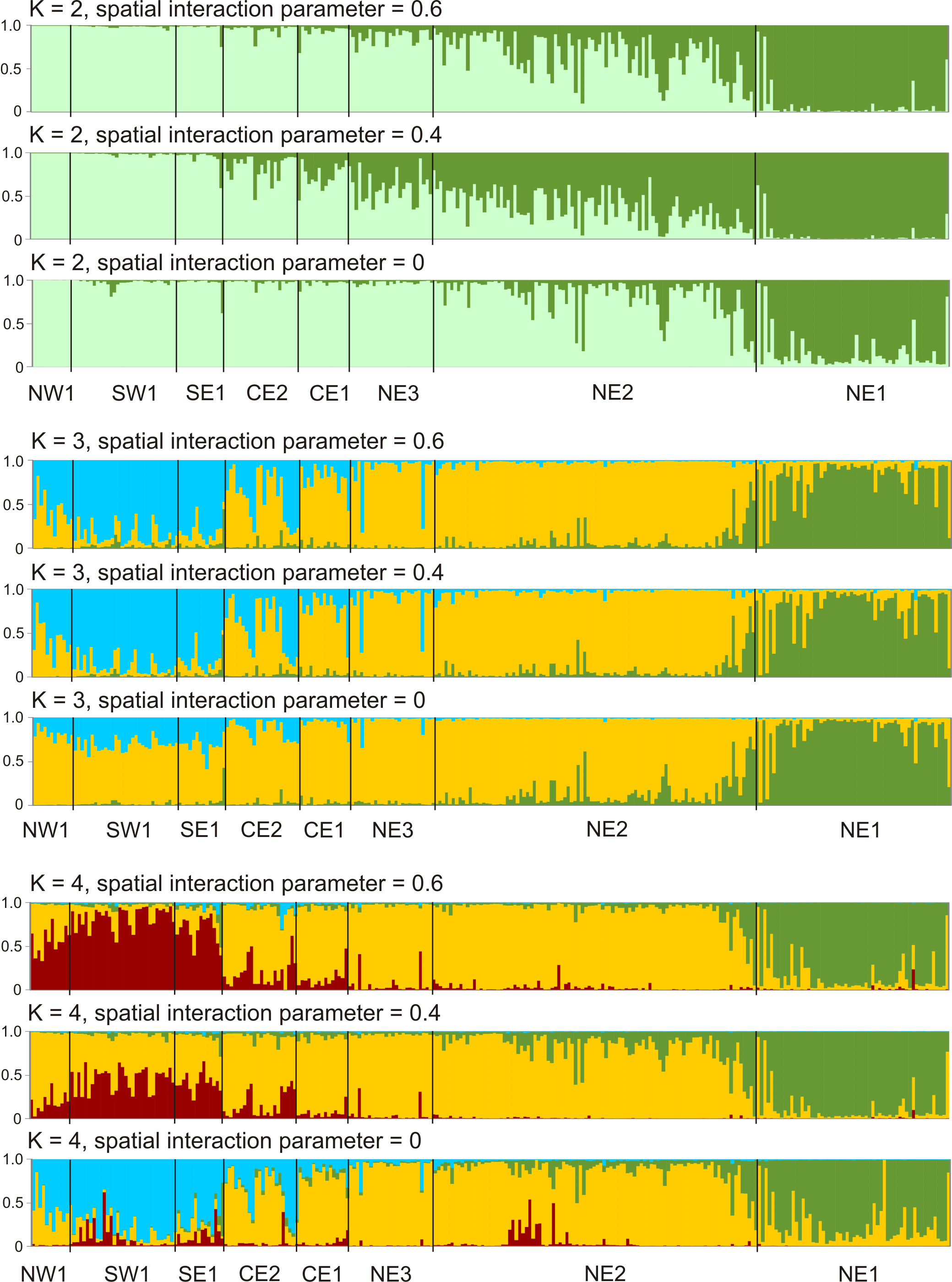
**

**Figure S4.** Genetic uniqueness index in relation to proportion of forests in 20 km buffers around each site in Poland.

**
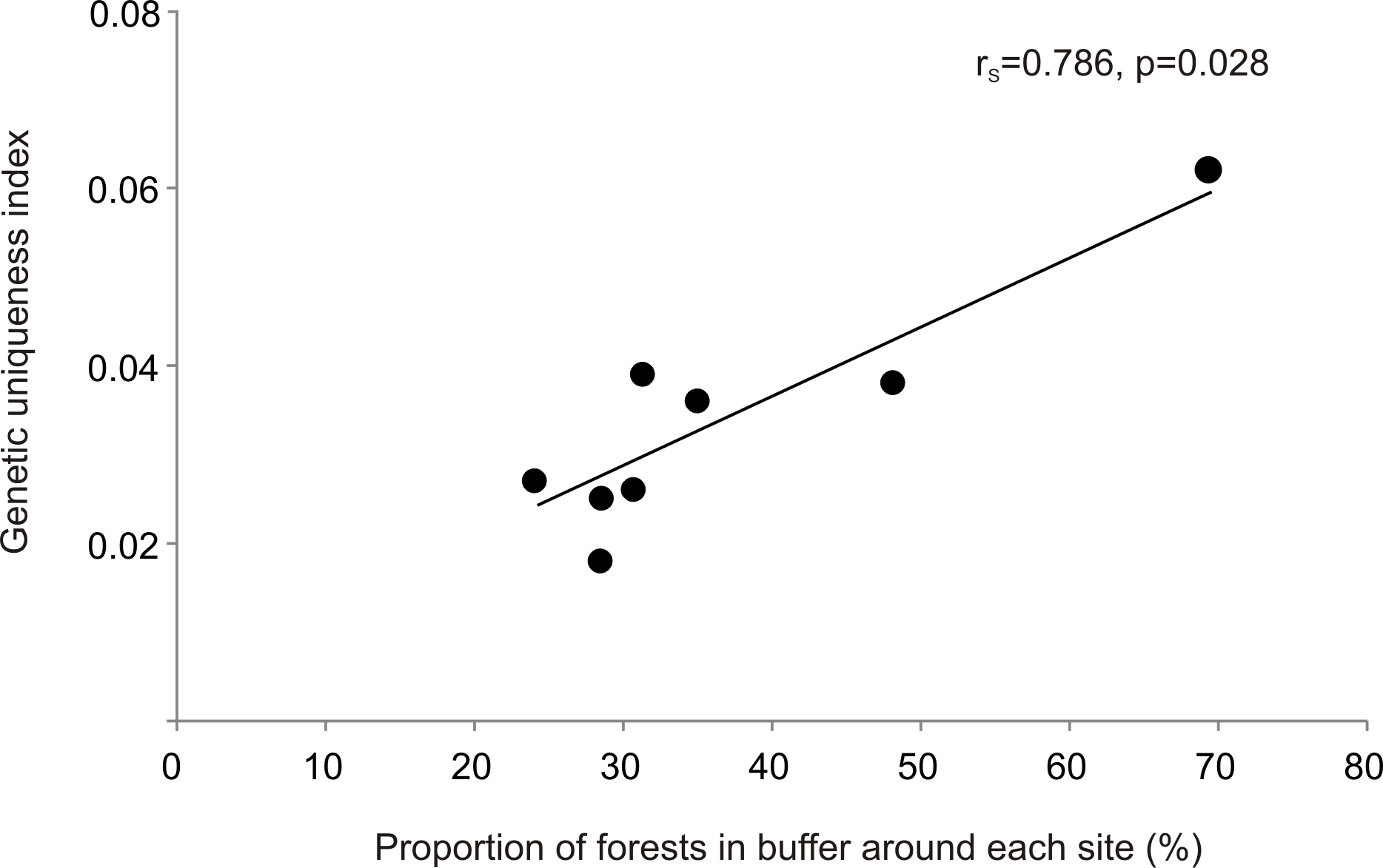
**

**Table S3.** Recent migration rate of stone marten between eight sites from Poland.

| Into/from |  | NE1 | NE2 | NE3 | CE1 | CE2 | SE1 | SW1 | CW1 |
| --- | --- | --- | --- | --- | --- | --- | --- | --- | --- |
| NE1 | Mean | **1** | 1.1e-10 | 1.1e-10 | 1.1e-10 | 1.1e-10 | 1.1e-10 | 1.1e-10 | 1.1e-10 |
|  | Mode | **1** | 2.4e-11 | 2.5e-11 | 2.4e-11 | 2.5e-11 | 2.5e-11 | 2.4e-11 | 2.4e-11 |
|  | (95% HDPI) | **(1;1)** | [4.4e-12;1.1e-09] | [4.9e-11;1.1e-09] | [1.9e-11;1.2e-09] | [3.9e-11;1.2e-09] | [2.5e-11;1.1e-09] | [4.2e-11;1.2e-09] | [3.4e-11;1.2e-09] |
| NE2 | Mean | 0.09 | **0.51** | 0.16 | 0.14 | 0.05 | 0.02 | 0.02 | 0.02 |
|  | Mode | 0.08 | **0.50** | 0.15 | 0.12 | 0.01 | 0.004 | 0.003 | 0.004 |
|  | (95% HDPI) | [4.8e-05;0.33] | **[0.21;0.74]** | [1.8e-05;0.42] | [5.9e-3;0.41] | [7.5e-3;0.34] | [4.8e-3;0.20] | [1.7e-3;0.14] | [4.9e-3;0.20] |
| NE3 | Mean | 4.7e-11 | 4.8e-11 | **1** | 4.7e-11 | 4.8e-11 | 4.8e-11 | 4.8e-11 | 4.8e-11 |
|  | Mode | 1.0e-11 | 1.1e-11 | **1** | 1.0e-11 | 1.0e-11 | 1.0e-11 | 1.0e-11 | 1.0e-11 |
|  | (95% HDPI) | [1.5e-11;4.8e-10] | [5.3e-12;5.2e-10] | **(1; 1)** | [1.7e-11;5.8e-10] | [3.6e-15;5.0e-10] | [1.7e-11;5.3e-10] | [1.0e-11;5.8e-10] | [1.0e-11;5.0e-10] |
| CE1 | Mean | 1.6e-10 | 1.6e-10 | 1.6e-10 | **1** | 1.6e-10 | 1.6e-10 | 1.6e-10 | 1.6e-10 |
|  | Mode | 2.6e-11 | 2.3e-11 | 2.6e-11 | **1** | 2.2e-11 | 2.6e-11 | 2.5e-11 | 2.3e-11 |
|  | (95% HDPI) | [5.7e-11;2.6e-09] | [5.6e-11;2.3e-09] | [1.0e-11;2.5e-09] | **(1; 1)** | [1.1e-11;2.7e-09] | [1.2e-10;2.6e-09] | [2.4e-11;2.4e-09] | [7.9e-11;2.3e-09] |
| CE2 | Mean | 2.7e-11 | 2.7e-11 | 2.6e-11 | 2.7e-11 | **1** | 2.7e-11 | 2.7e-11 | 2.7e-11 |
|  | Mode | 6.4e-12 | 6.2e-12 | 6.4e-12 | 6.4e-12 | **1** | 6.4e-12 | 6.3e-12 | 6.3e-12 |
|  | (95% HDPI) | [1.4e-12;1.4e-10] | [9.3e-13;1.5e-10] | [2.0e-12;1.7e-10] | [6.05e-12;1.6e-10] | **(1; 1)** | [2.1e-12;1.5e-10] | [4.6e-12;1.4e-10] | [5.2e-12;1.4e-10] |
| SE1 | Mean | 2.1e-10 | 2.0e-10 | 2.1e-10 | 2.0e-10 | 2.0e-10 | **1** | 2.1e-10 | 2.1e-10 |
|  | Mode | 4.8e-11 | 4.9e-11 | 4.9e-11 | 4.9e-11 | 4.9e-11 | **1** | 4.8e-11 | 4.7e-11 |
|  | (95% HDPI) | [4.2e-11;1.2e-09] | [5.1e-11;1.2e-09] | [4.2e-11;1.2e-09] | [4.2e-11;1.2e-09] | [2.7e-11;1.1e-09] | **(1; 1)** | [9.6e-15;1.1e-09] | [2.5e-15;1.1e-09] |
| SW1 | Mean | 6.8e-10 | 6.8e-10 | 6.82e-10 | 6.7e-10 | 6.7e-10 | 6.8e-10 | **1** | 6.7e-10 |
|  | Mode | 1.2e-10 | 1.2e-10 | 1.3e-10 | 1.2e-10 | 1.2e-10 | 1.3e-10 | **1** | 1.2e-10 |
|  | (95% HDPI) | [2.4e-10;8.4e-09] | [4.1e-10;9.1e-09] | [9.1e-11;8.9e-09] | [4.8e-10;9.9e-09] | [3.5e-10;8.9e-09] | [3.9e-10;8.2e-09] | **(1; 1)** | [2.3e-10;8.7e-09] |
| NW1 | Mean | 2.1e-11 | 2.2e-11 | 2.2e-11 | 2.2e-11 | 2.1e-11 | 2.1e-11 | 2.1e-11 | **1** |
|  | Mode | 5.0e-12 | 5.0e-12 | 5.3e-12 | 5.0e-12 | 5.0e-12 | 5.0e-12 | 5.1e-12 | **1** |
|  | (95% HDPI) | [5.0e-12;1.2e-10] | [3.3e-12;1.2e-10] | [4.7e-12;1.4e-10] | [3.2e-12;1.2e-10] | [5.0e-12;1.2e-10] | [5.7e-12;1.2e-10] | [4.1e-12;1.2e-10] | **(1; 1)** |

Estimations are based on posterior means and modes calculated by BIMR. Within-site movement is marked in bold.

**Table S4.** Results from the BOTTLENECK tests for detection of recent reduction or expansion in effective population size for eight sites of stone marten (*Martes foina*) in Poland.

| Site | N | Model | | | | | | | | | |  | Mode shift |
| --- | --- | --- | --- | --- | --- | --- | --- | --- | --- | --- | --- | --- | --- |
|  |  | SMM | |  | TPM: 0.22, 0.12 | | |  | TPM: 0.05, 0.12 | | |  |  |
|  |  | Sign test | Wilcoxon test |  | Sign test | Wilcoxon test | |  | Sign test | Wilcoxon test | |  |  |
|  |  |  |  |  |  | H excess | H deficiency |  |  | H excess | H deficiency |  |  |
| NE1 | 58 | 0.530 | 0.636 |  | 0.302 | 0.108 | 0.899 |  | 0.341 | 0.449 | 0.565 |  | L |
| NE2 | 95 | 0.132 | 0.794 |  | 0.226 | 0.078 | 0.926 |  | 0.581 | 0.486 | 0.527 |  | L |
| NE3 | 25 | 0.568 | 0.607 |  | 0.400 | 0.196 | 0.813 |  | 0.576 | 0.500 | 0.514 |  | **Shifted mode** |
| CE1 | 15 | 0.127 | 0.216 |  | 0.247 | 0.187 | 0.822 |  | 0.111 | 0.620 | 0.393 |  | **Shifted mode** |
| CE2 | 22 | 0.424 | 0.406 |  | 0.215 | 0.051 | 0.952 |  | 0.250 | 0.270 | 0.742 |  | L |
| SE1 | 14 | 0.311 | 0.594 |  | 0.302 | 0.270 | 0.741 |  | 0.497 | 0.459 | 0.554 |  | L |
| SW1 | 31 | 0.224 | 0.527 |  | 0.251 | **0.048** | 0.956 |  | 0.386 | 0.406 | 0.607 |  | L |
| NW1 | 12 | 0.240 | 0.753 |  | 0.568 | 0.367 | 0.646 |  | 0.519 | 0.607 | 0.406 |  | L |

For the two-phase model (TPM) parameters given are the proportion of multi-step mutations and the mean size of multi-step mutations. For the mode shift method, L refers to the pattern expected in the absence of a recent reduction in size. SMM, stepwise mutation model. Statistically significant values (p<0.05) are marked in bold. N – sample size.

**Table S5.** Point estimates and 95% confidence intervals (in parentheses) of the demographic parameters estimated with MIGRAINE analyses of stone marten populations, under OnePopVarSize demographic model.

| Site | pGSM | 2Nµ | 2N*_anc_*µ | Dg/2N | Dg*µ | N*_ratio_* | Demography |
| --- | --- | --- | --- | --- | --- | --- | --- |
| NE1 | 0.35  (0.14–0.49) | 0.32  (0.046 - 0.68) | 1.8  (0.84 - 5.3) | 0.36  (0.051 - 1.2) | 0.12  (0.0053 - 0.66) | 0.18  (0.032 - 0.62) | Significant contraction |
| NE2 | 0.29  (0.13 - 0.43) | 0.60  (0.27 - 1.0) | 2.2  (1.1 - 5.5) | 0.36  (0.061 - 1.4) | 0.21  (0.020 - 1.2) | 0.28  (0.098 - 0.67) | Significant contraction |
| NE3 | 0.27  (0.11 - 0.41) | 0.60  (9.0e - 08 - 1.2) | 2.3  (0.62 - 71) | 0.32  (0.030 - 10) | 0.19  (0.022 - 3.6) | 0.26  (4.3e - 05 - 1.9) | Almost significant contraction |
| CE1 | 0.38  (0.26 - 0.50) | 1.3  (0.97 – 1.8) | NA | NA | NA | 0.0805  (0.000262 - 1038) | Stable population, non-significant contraction signal |
| CE2 | 0.36  (0.24 - 0.48) | 1.2  (0.88 – 1.6) | N A | NA | NA | 0.293  (0.000151 - 488) | Stable population, non-significant contraction signal |
| CE1+CE2 | 0.31  (0.17 – 0.47) | 0.022  (0.000001 – 0.96) | 1.8  (1.1 – 3.3) | 0.14  (0.035 – 0.98) | 0.0030  (1.4e-7 – 0.42) | 0.012  (2.5e-7 – 0.60) | Significant contraction |
| SE1 | 0.28  (0.15 – 0.4) | 1.6  (1.2 – 2.2) | N A | NA | NA | 0.25  (3.6e-7 – 180) | Stable population |
| SW1 | 0.30  (0.18 – 0.42) | 1.3  (1.0 – 1.8) | N A | NA | NA | 0.11  (4.8e-7 - 230) | Stable population |
| NW1 | 0.27  (0.14 – 0.41) | 1.3  (0.91 – 1.8) | N A | NA | NA | 0.099  (2.8e-7 – 7.7) | Stable population |

Unscaled parameters, computed for a mutation rate of 0.0005

| Site | N  (individuals) | N*_anc_*  (individuals) | Dg from Dg/2N  (generations) | Dg from Dg* µ  (generations) |
| --- | --- | --- | --- | --- |
| NE1 | 160  (23 - 340) | 880  (420 - 2,800) | 233  (5 - 1,600) | 232  (11 - 1,300) |
| NE2 | 300  (135 - 504) | 1100  (540 - 2,800) | 427  (33 - 2,800) | 426  (40 - 2,400) |
| NE3 | 298  (1 - 620) | 1200  (312 - 35,000) | 380  (1 - 26,000) | 380  (44 - 7,100) |
| CE1 | 650  (490 – 900) | NA | NA | NA |
| CE2 | 600  (440 - 800) | NA | NA | NA |
| CE1+CE2 | 11  (1 - 480) | 920  (560 - 1600) | 6  (1 - 1900) | 6  (1 - 830) |
| SE1 | 800  (600 – 1100) | N A | N A | NA |
| SW1 | 650  (500 – 900) | N A | N A | NA |
| NW1 | 650  (460 – 900) | N A | N A | NA |

**Table S6.** Point estimates and 95% confidence intervals (in parentheses) of the demographic parameters estimated with MIGRAINE analyses of stone marten populations, under OnePopFounderFlush demographic model.

| Site | 2Nµ | Dg/2N | Dg* µ | 2N*_founder_*µ | 2N*_anc_*µ | N*_anc ratio_* | N*_cur_*N*_founder_* _ratio_ | N*_founder_*N*_anc ratio_* | Demography |
| --- | --- | --- | --- | --- | --- | --- | --- | --- | --- |
| NE1 | 18.99  (0.194-NA) | 0.000433  (4.2e-06-0.425) | 0.00823  (0.00195-0.168) | 0.00945  (0.00145-NA) | 2.078  (1.046-4.874) | 9.139  (0.0809-425.4) | 2009  (0.191-276684) | 0.00455  (0.00055-0.575) | Founder event, without expansion |
| NE2+NE3 | 8.838  (1.465-438.1) | 0.00253  (3.29e-05-0.0417) | 0.0224  (0.0088-0.0711) | 0.0483  (0.0113-0.25) | 2.088  (1.209-3.896) | 4.233  (0.609-241.7) | 183.1  (6.502-26310) | 0.0231  (0.00478-0.142) | Founder event and expansion |
| CE1+CE2 | 1.272  (NA-NA) | 2.67e-0  (NA-0.313) | 3.4e-06  (4.99e-07-0.183) | 1.14e-05  (3.36e-06-7.14) | 1.853  (1.196-3.049) | 0.686  (0.00318-860) | 111487  (0.00164-993569) | 6.16e-06  (NA-5.531) | Stable population |

Unscaled parameters, computed for a mutation rate of 0.0005

| Site | N  (individuals) | N*_founder_*  (individuals) | N*_anc_*  (individuals) | Dg from Dg/2N  (generations) | Dg from Dg* µ  (generations) |
| --- | --- | --- | --- | --- | --- |
| NE1 | 9435  (85 – NA) | 5  (1 – NA) | 1039  (518 - 2260) | 17  (1 – NA) | 17  (4 - 336) |
| NE2+NE3 | 4419  (733 – 219,050) | 24  (6 - 125) | 1044  (605 - 1948) | 45  (1 – 36538) | 45  (18 - 142) |
| CE1+CE2 | 636  (NA – NA) | NA | 926  (598 - 1524) | NA | NA |

**Figure S5.** Pairwise likelihood-ratio profiles obtained with Migraine under the OnePopFounderFlush model for central sites (pooled CE1 and CE2), north-eastern sites (pooled NE2 and NE3) and site NE1 from Poland.

**
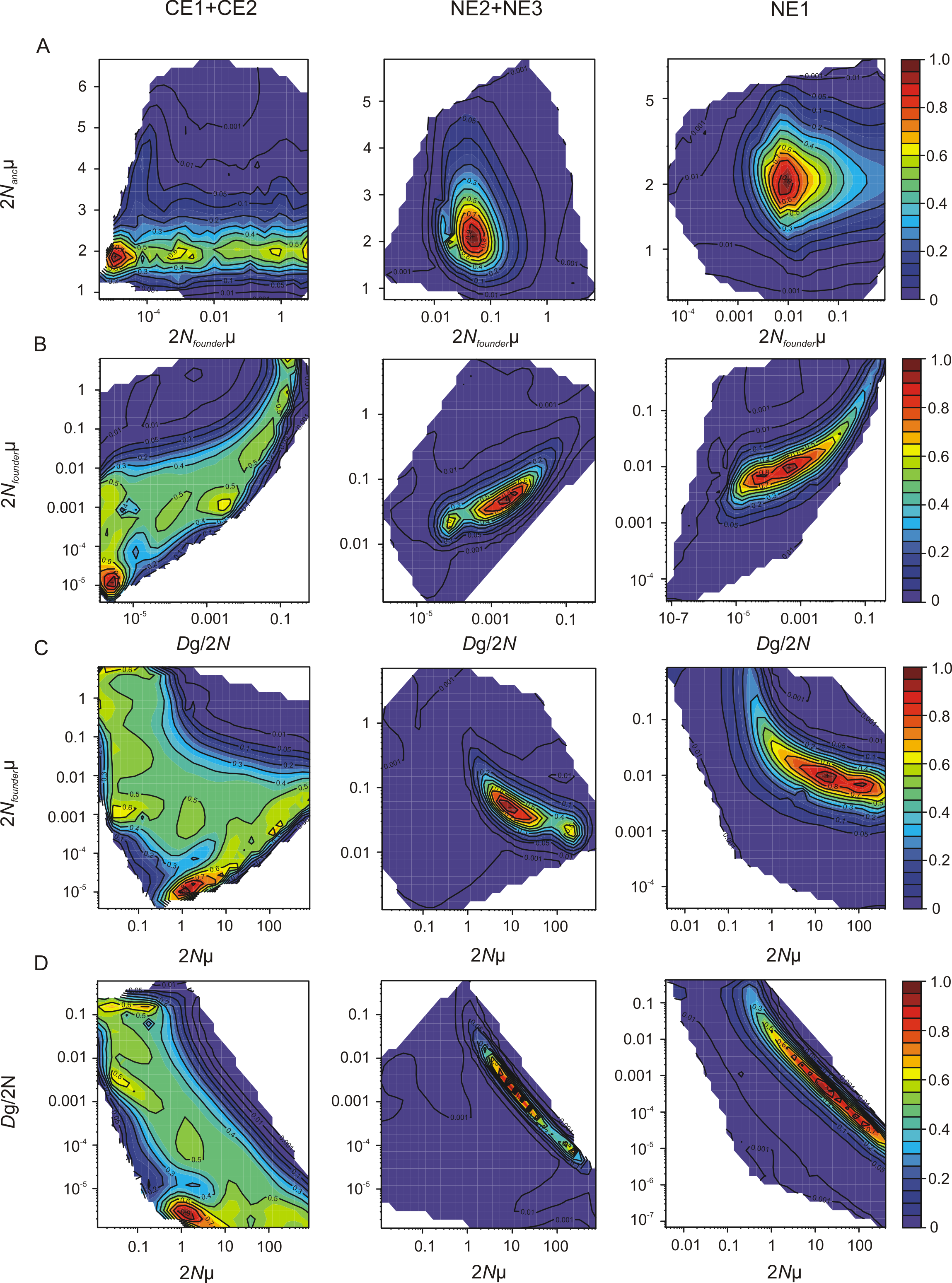
**

Pairwise likelihood-ratio profiles obtained for some pairs of the following parameters: 2*N*_anc_µ: ancestral effective population size; 2*N*_founder_µ: founder population size; 2*N*µ: current effective population size and *D*: timing of the demographic history events. Very recent founder events (panel A and B) were detected for NE2+NE3 and NE1 and significant expansion (panel C) for NE2+NE3, contrasting with a stable population for sites CE1+CE2. All axes are represented using a log scale. Point estimate values for each parameter and the associated 95% confidence interval are shown in Table S10. Pairwise likelihood-ratio profiles are computed from full-parameter likelihood surfaces by maximization of all other parameters, and subsequently computing log-likelihood ratio p-vales of each point by the χ^2^ method, in the same way as generic likelihood confidence intervals. For example, a two-dimensional confidence region for (2*N*_anc_**μ, 2*N*_founder_**μ) is deduced by comparing the profile likelihood ratio (actually, twice its logarithm, i.e. 2{ln[L(θ)] − ln[L(θ)]}) to the χ^2^ distribution with 2 d. Likelihood profile are inferred only from the likelihood of parameters restricted within the convex hull of sampled parameter points (the colored part of the graphic), i.e. ignoring values inferred by the Kriging prediction outside this region.
