# Supplementary material for: Genetic diversity and structure related to expansion history and habitat isolation: stone marten populating rural–urban habitats
Source: BMC Ecol. 2017 Dec 22;17:46. doi: 10.1186/s12898-017-0156-6 (PMC5741947; doi:10.1186/s12898-017-0156-6)
Supplement: Supplementary file 2 — Additional file 2. Row experimental data. [file 12898_2017_156_MOESM2_ESM.pdf]

Number	SITE	Ma8	Ma8	Lut615	Lut615	Lut27	Lut27	Mp059	Mp059	Mf3.2	Mf3.2	Mf4.10	Mf4.10
034km	A_NE1	113	113	246	246	187	187	145	151	156	156	311	311
111km	A_NE1	113	117	246	246	187	187	147	151	152	156	327	339
029F	A_NE1	113	117	246	246	187	187	147	151	156	164	311	339
154km	A_NE1	113	117	242	246	187	191	151	151	152	156	311	311
054F	A_NE1	113	117	242	246	187	187	151	151	156	156	311	331
022M	A_NE1	113	113	242	246	187	191	151	151	156	160	311	339
043F	A_NE1	117	117	242	246	191	191	147	151	156	160	311	319
004M	A_NE1	117	117	246	246	187	187	151	151	152	156	311	315
031M	A_NE1	117	117	242	246	187	187	151	151	156	164	311	319
025M(17M)	A_NE1	113	117	246	246	187	191	147	151	152	152	311	339
029M	A_NE1	113	117	246	246	187	187	151	151	152	164	311	319
369km	A_NE1	113	117	246	246	187	191	151	151	156	156	311	327
021F	A_NE1	117	117	242	246	187	187	151	151	152	152	311	311
052F(40F)	A_NE1	113	117	242	246	187	191	151	151	152	164	311	319
025F	A_NE1	113	117	244	246	187	187	147	151	152	164	311	311
024F(289)	A_NE1	113	113	246	246	187	187	147	151	152	164	311	319
035M	A_NE1	113	117	242	246	187	187	151	151	156	156	311	327
198km	A_NE1	113	117	244	246	187	191	147	151	152	164	311	311
026M	A_NE1	113	117	242	246	187	187	151	151	152	164	315	339
032M	A_NE1	113	117	246	246	187	191	151	151	152	156	319	327
110km	A_NE1	113	113	246	246	187	191	151	151	156	156	311	323
006_7M	A_NE1	113	117	242	246	187	191	151	151	156	156	311	311
002F	A_NE1	113	113	246	246	191	195	151	151	152	156	311	311
002km	A_NE1	117	117	242	246	187	191	151	151	152	164	311	327
008F	A_NE1	113	117	246	246	187	187	151	151	152	156	315	327
039F	A_NE1	113	113	242	246	191	191	151	151	156	160	327	339
197km	A_NE1	113	113	242	246	187	191	147	151	152	160	311	327
087km	A_NE1	113	113	246	246	187	187	151	151	152	156	311	327
009M	A_NE1	117	117	242	246	187	187	151	151	156	156	327	327
007M	A_NE1	113	117	246	246	187	187	151	151	152	156	311	311
001M	A_NE1	113	113	246	246	187	187	151	151	152	152	315	315
013km	A_NE1	113	117	246	246	187	187	151	151	152	152	311	315

Number	SITE	Ma8	Ma8	Lut615	Lut615	Lut27	Lut27	Mp059	Mp059	Mf3.2	Mf3.2	Mf4.10	Mf4.10
031F	A_NE1	117	117	242	246	187	191	151	151	152	156	311	311
057km(5M)	A_NE1	113	117	242	246	187	187	151	151	156	156	315	327
047F	A_NE1	113	113	246	246	187	191	151	151	156	164	311	311
016M	A_NE1	113	117	242	246	187	187	151	151	152	156	311	327
106km	A_NE1	113	117	246	246	191	191	147	151	164	164	311	327
042F	A_NE1	113	113	242	242	187	191	151	151	156	164	311	323
023F	A_NE1	113	113	242	246	187	187	151	151	152	156	311	323
006M	A_NE1	113	113	246	246	187	191	151	151	152	156	311	327
050F	A_NE1	113	113	242	246	191	191	147	151	152	164	311	319
325km	A_NE1	113	113	242	242	187	191	147	151	156	160	311	327
38F	A_NE1	113	117	242	246	187	191	151	151	152	156	315	327
019M	A_NE1	113	113	242	242	187	187	151	151	152	156	311	323
049F	A_NE1	113	113	246	246	187	191	151	151	152	156	311	311
023M	A_NE1	113	113	242	242	187	187	151	151	156	156	311	339
090km	A_NE1	113	113	242	242	187	187	151	151	152	152	311	315
033M	A_NE1	113	117	246	246	187	191	151	151	156	156	327	327
028F	A_NE1	113	113	246	246	187	191	147	151	152	164	311	311
207km	A_NE1	117	117	246	246	187	191	151	151	156	164	311	327
055F	A_NE1	113	117	242	242	187	191	151	151	156	164	319	327
022km	A_NE1	113	119	246	246	187	191	147	151	156	164	311	315
056km	A_NE1	113	117	242	246	187	195	151	151	156	156	311	315
123km	A_NE1	113	117	242	246	187	187	151	151	152	152	311	311
113km	A_NE1	113	113	242	246	187	195	151	151	156	156	311	315
348km	A_NE1	113	113	246	246	187	191	151	151	152	156	311	311
112km	A_NE1	113	117	240	242	187	195	151	151	152	156	311	315
203km	A_NE1	113	117	242	246	187	191	147	151	156	156	311	311
450km	B_NE2	113	117	242	246	187	191	151	151	152	152	311	327
499km	B_NE2	113	117	242	246	187	191	147	147	156	156	311	311
296km	B_NE2	113	117	242	246	187	187	151	151	156	164	319	327
349km	B_NE2	113	117	246	246	187	187	151	151	156	156	319	319
354km	B_NE2	113	117	242	246	187	187	151	151	156	156	315	331
225km	B_NE2	113	117	246	246	187	187	151	151	152	164	311	315

Number	SITE	Ma8	Ma8	Lut615	Lut615	Lut27	Lut27	Mp059	Mp059	Mf3.2	Mf3.2	Mf4.10	Mf4.10
316km	B_NE2	117	117	242	242	187	187	151	151	148	164	319	327
396km	B_NE2	113	117	246	246	187	191	151	151	156	156	311	319
315km	B_NE2	113	113	242	242	187	187	151	151	156	156	311	315
223km	B_NE2	117	117	246	246	187	187	147	151	152	152	311	311
045F	B_NE2	113	117	246	246	187	195	151	151	152	156	311	331
003km	B_NE2	113	117	242	246	187	187	147	151	156	160	311	315
393km	B_NE2	113	117	242	246	187	191	147	151	152	156	311	327
018M	B_NE2	117	117	246	246	191	191	151	151	156	156	311	327
046F	B_NE2	113	117	242	246	187	187	147	151	156	156	311	311
20F	B_NE2	117	117	242	246	187	191	147	151	156	156	315	327
028M	B_NE2	117	117	242	242	187	191	151	151	152	152	311	311
034M	B_NE2	113	117	246	246	187	195	151	151	152	156	319	331
226km	B_NE2	113	117	246	246	187	187	147	151	156	156	311	327
022F	B_NE2	113	113	240	242	187	191	147	151	152	156	311	327
037F	B_NE2	113	117	242	246	187	187	151	151	152	156	311	311
053F	B_NE2	113	117	242	246	187	191	151	151	156	156	311	315
020M	B_NE2	113	117	242	244	187	191	151	153	152	156	311	319
493km	B_NE2	113	113	246	246	187	191	151	151	152	156	315	319
536km	B_NE2	117	117	246	246	187	191	151	151	152	156	311	311
021M(451)	B_NE2	113	113	246	246	187	187	151	151	156	156	311	315
051F	B_NE2	113	113	246	246	187	191	151	151	164	164	311	315
044F	B_NE2	113	113	246	246	187	187	151	151	156	164	311	315
027M	B_NE2	113	113	244	246	187	187	151	151	156	156	311	315
239km	B_NE2	113	117	242	246	187	187	147	151	152	152	311	311
368km	B_NE2	117	117	246	246	187	195	151	151	156	156	311	315
048F	B_NE2	113	117	242	246	187	187	151	151	156	164	311	331
147km	B_NE2	113	117	246	246	187	187	151	151	152	156	319	327
034F	B_NE2	113	113	246	246	187	187	151	151	156	164	311	311
116km	B_NE2	117	117	242	246	187	187	151	151	152	156	311	315
210km	B_NE2	113	113	246	246	187	187	147	151	156	164	311	319
405km	B_NE2	117	117	244	246	187	187	151	151	156	164	323	331
323km	B_NE2	113	113	242	244	187	187	149	153	152	156	315	331

Number	SITE	Ma8	Ma8	Lut615	Lut615	Lut27	Lut27	Mp059	Mp059	Mf3.2	Mf3.2	Mf4.10	Mf4.10
476km	B_NE2	113	117	242	246	187	187	151	151	152	156	315	331
297km	B_NE2	113	117	242	246	187	187	145	151	156	156	319	327
324km	B_NE2	113	117	246	246	187	187	151	151	156	156	311	327
241km	B_NE2	117	117	246	246	187	187	147	151	152	156	311	311
217km	B_NE2	117	117	240	242	187	195	151	151	152	152	315	327
138km	B_NE2	113	113	242	246	187	195	151	151	152	164	311	315
455km	B_NE2	117	117	242	246	187	187	151	153	156	156	311	319
211km	B_NE2	113	117	242	246	187	187	147	151	156	156	311	311
222km	B_NE2	113	113	240	246	187	191	151	151	152	156	311	331
108km	B_NE2	117	117	242	246	187	191	151	151	152	152	311	319
085km	B_NE2	117	117	244	246	187	191	151	153	156	156	311	327
127km	B_NE2	113	117	240	246	187	187	151	151	152	156	319	319
044km	B_NE2	113	113	242	246	187	187	145	151	156	156	311	311
282km	B_NE2	113	113	246	246	187	187	151	151	152	156	315	319
403km	B_NE2	113	113	246	246	191	191	145	147	152	156	311	311
365km	B_NE2	113	117	242	246	187	187	147	151	156	164	311	311
371km	B_NE2	113	117	246	246	187	187	147	151	152	160	315	327
137km	B_NE2	113	119	246	246	187	191	147	147	152	152	311	311
299km	B_NE2	113	117	242	246	187	187	151	151	156	156	311	315
362-2km	B_NE2	113	113	246	246	187	195	145	151	156	156	311	315
308km	B_NE2	113	117	242	242	191	195	147	151	156	164	311	315
140km	B_NE2	113	113	240	244	187	187	147	151	140	164	311	315
238km	B_NE2	113	113	244	246	187	187	147	151	152	156	311	315
402km	B_NE2	113	113	246	246	187	187	151	151	152	152	311	323
397km	B_NE2	113	113	246	246	187	187	151	151	156	156	319	319
270km	B_NE2	113	117	240	242	187	195	147	147	156	164	315	319
276km	B_NE2	113	117	242	244	187	187	147	151	156	156	311	319
474km	B_NE2	113	117	240	242	187	187	147	151	156	156	311	315
220_7km	B_NE2	113	113	246	246	195	195	147	151	152	156	311	311
220_6km	B_NE2	113	117	242	246	187	187	151	151	156	156	311	331
220_2km	B_NE2	113	113	242	246	191	195	147	147	152	156	311	315
220_4km	B_NE2	113	117	242	246	187	195	147	151	152	156	311	319

Number	SITE	Ma8	Ma8	Lut615	Lut615	Lut27	Lut27	Mp059	Mp059	Mf3.2	Mf3.2	Mf4.10	Mf4.10
220_3km	B_NE2	117	117	240	242	187	187	151	151	156	160	311	319
220_1km	B_NE2	113	113	246	246	187	195	147	147	152	156	311	315
267km	B_NE2	113	117	242	242	187	191	147	151	156	156	315	315
372km	B_NE2	113	117	242	246	187	191	151	151	156	164	315	315
287km	B_NE2	113	117	244	246	187	195	151	151	156	156	315	323
286km	B_NE2	113	113	242	246	187	187	147	151	156	156	311	315
284km	B_NE2	113	117	242	242	187	187	147	151	152	152	319	327
283km	B_NE2	113	117	242	246	187	187	151	151	152	156	311	315
281km	B_NE2	113	117	242	242	187	195	145	151	156	156	323	323
292km	B_NE2	117	117	246	246	187	187	151	151	156	156	315	327
295km	B_NE2	113	117	242	246	187	187	147	151	152	156	311	319
234km	B_NE2	113	113	246	246	187	187	147	147	152	156	311	315
377km	B_NE2	117	117	246	246	191	195	151	151	152	156	315	315
437km	B_NE2	113	113	242	246	187	187	151	151	152	156	311	315
229km	B_NE2	113	113	242	246	187	187	151	151	152	152	327	327
269km	B_NE2	113	117	242	242	187	191	151	151	156	156	0	0
357km	B_NE2	113	117	246	246	187	191	151	151	156	164	315	315
358km	B_NE2	113	117	242	246	187	187	151	151	156	160	311	315
237km	B_NE2	113	113	246	246	187	195	151	151	152	156	315	323
376km	B_NE2	117	117	246	246	187	191	151	151	140	152	315	319
503km	B_NE2	113	117	244	246	187	191	147	151	156	160	315	323
367km	B_NE2	113	113	242	242	187	191	147	151	156	164	315	327
404km	B_NE2	113	113	242	246	187	191	151	151	156	164	315	319
406km	B_NE2	113	117	246	246	187	191	151	151	152	164	315	319
208km	B_NE2	117	117	242	242	187	195	151	151	152	156	319	327
522km	C_NE3	113	113	242	246	187	187	151	151	156	156	319	323
446km	C_NE3	113	113	244	246	187	187	151	151	152	156	319	319
543km	C_NE3	113	117	246	246	187	187	147	151	152	156	311	327
353_1km(PC	C_NE3	113	117	242	246	187	191	147	151	152	156	315	319
527km	C_NE3	117	117	246	246	187	187	151	151	152	156	311	327
448km	C_NE3	117	117	246	246	187	187	151	151	144	156	311	311
274km	C_NE3	117	117	242	242	187	191	145	151	152	156	311	311

Number	SITE	Ma8	Ma8	Lut615	Lut615	Lut27	Lut27	Mp059	Mp059	Mf3.2	Mf3.2	Mf4.10	Mf4.10
311km	C_NE3	113	117	246	246	187	191	151	151	152	152	319	323
248km	C_NE3	117	117	242	242	187	187	151	151	152	156	315	315
244km	C_NE3	113	117	246	246	187	195	151	151	152	156	315	327
443km	C_NE3	113	113	244	246	187	187	151	151	152	156	311	319
246km	C_NE3	113	117	242	246	187	187	151	151	160	160	311	327
440km	C_NE3	113	113	242	242	191	195	147	151	152	156	311	327
442km	C_NE3	113	113	246	246	187	195	151	151	152	156	311	323
441km	C_NE3	113	117	242	246	187	187	151	151	156	156	315	327
179km	C_NE3	113	117	246	246	187	187	151	151	152	156	315	323
245-akm	C_NE3	113	117	244	246	187	187	151	151	156	156	319	327
245km	C_NE3	113	113	242	246	187	187	147	151	156	156	311	315
232_1km	C_NE3	113	117	244	244	187	187	151	151	156	156	311	327
232_2km	C_NE3	113	117	242	246	187	187	151	151	156	164	311	323
436km	C_NE3	113	113	246	246	187	187	147	151	152	156	311	327
249km	C_NE3	117	117	242	246	197	199	151	151	156	156	311	323
243km	C_NE3	113	113	246	246	187	191	151	151	152	156	319	327
247km	C_NE3	117	117	242	246	187	191	151	151	156	156	311	327
449km	C_NE3	113	117	244	246	187	191	151	151	152	156	315	315
086km	D_CE1	117	117	242	246	187	189	145	147	152	156	319	319
391km	D_CE1	113	117	242	246	187	195	151	151	136	140	315	319
169km	D_CE1	113	117	240	246	187	191	147	151	152	156	311	319
326km	D_CE1	113	117	246	246	187	195	151	151	152	152	315	327
300km	D_CE1	113	113	242	246	187	187	147	151	152	156	311	315
411km	D_CE1	113	113	240	246	187	187	151	151	156	156	311	323
115km(PCR1	D_CE1	113	117	246	246	187	187	147	149	156	164	315	327
395km	D_CE1	113	113	246	246	191	195	151	151	156	156	319	323
305km	D_CE1	113	117	242	246	187	187	151	151	156	156	319	319
167km	D_CE1	113	117	242	242	187	195	147	151	152	156	319	323
519km	D_CE1	113	117	242	244	191	195	151	151	156	156	315	323
475km	D_CE1	117	117	242	246	187	191	147	151	156	156	315	327
242km	D_CE1	113	113	240	242	187	187	145	151	152	156	319	319
228km	D_CE1	117	117	240	242	187	187	145	151	140	152	315	319

Number	SITE	Ma8	Ma8	Lut615	Lut615	Lut27	Lut27	Mp059	Mp059	Mf3.2	Mf3.2	Mf4.10	Mf4.10
212km	D_CE1	113	117	246	246	187	191	145	147	156	156	315	339
107km	E_CE2	113	117	242	246	187	187	151	151	156	156	327	327
484km	E_CE2	113	117	246	246	187	191	147	151	152	156	315	319
480km	E_CE2	113	117	242	244	187	191	151	151	152	156	311	319
481km	E_CE2	117	117	242	242	187	187	151	151	152	156	311	319
398km	E_CE2	117	117	242	246	195	195	151	151	156	156	315	327
252km	E_CE2	117	117	242	242	187	191	151	151	152	156	315	327
253km	E_CE2	117	117	242	244	187	187	151	151	152	156	315	315
236km	E_CE2	113	117	242	246	187	191	151	151	152	156	315	319
260km	E_CE2	117	117	242	242	187	187	151	151	156	156	331	331
250km	E_CE2	113	113	242	242	187	187	145	151	152	156	311	319
262km	E_CE2	113	117	242	242	187	191	151	151	152	152	319	339
265km	E_CE2	113	117	246	246	187	195	151	151	156	156	315	315
168km	E_CE2	113	113	242	246	191	191	147	147	156	164	319	323
258km	E_CE2	117	117	242	242	187	189	151	151	156	156	311	311
255km	E_CE2	117	117	242	244	187	195	151	151	152	156	311	331
256km	E_CE2	113	117	242	246	187	187	151	151	152	156	315	315
264km	E_CE2	117	117	242	244	187	195	151	151	152	152	315	319
251km	E_CE2	113	117	242	242	187	187	147	147	152	152	315	319
261km	E_CE2	113	117	246	246	187	187	147	151	152	156	319	319
257km	E_CE2	113	113	242	242	187	187	151	151	152	156	315	315
254km	E_CE2	113	113	242	246	187	187	147	151	152	156	311	319
263km	E_CE2	117	117	242	242	187	187	147	153	152	156	315	339
333km	F_SE1	113	117	242	244	187	187	151	151	156	164	315	319
29Wr	F_SE1	113	113	242	246	187	191	147	151	136	156	311	319
386km	F_SE1	117	119	240	246	191	191	147	151	152	152	315	315
388km	F_SE1	113	117	242	246	187	195	147	151	156	156	311	319
383km	F_SE1	117	117	244	244	187	187	145	151	152	156	311	327
381km	F_SE1	113	117	244	246	187	195	145	149	156	156	319	319
385km	F_SE1	117	117	244	246	187	187	145	151	152	152	311	311
382km	F_SE1	117	117	242	244	187	195	147	151	152	156	319	319
380km	F_SE1	113	117	246	246	191	195	145	151	152	156	311	311

Number	SITE	Ma8	Ma8	Lut615	Lut615	Lut27	Lut27	Mp059	Mp059	Mf3.2	Mf3.2	Mf4.10	Mf4.10
384km	F_SE1	117	117	244	246	187	195	151	153	156	156	315	319
390km	F_SE1	117	117	246	246	187	195	151	151	156	160	315	327
387km	F_SE1	117	117	246	246	191	195	151	151	156	156	311	311
389km	F_SE1	117	117	240	242	187	187	151	151	156	156	311	319
378km	F_SE1	113	117	242	246	187	195	151	151	152	156	311	327
1Wr	G_SW1	113	117	244	246	187	187	147	147	156	156	319	327
16Wr	G_SW1	117	117	246	246	187	195	147	151	152	156	319	319
11Wr	G_SW1	117	117	246	246	191	195	151	151	152	156	311	311
6Wr	G_SW1	113	117	240	246	191	195	147	151	156	156	315	319
15Wr	G_SW1	117	117	246	246	187	191	147	151	152	156	319	319
13Wr	G_SW1	117	119	246	246	187	191	151	151	156	156	319	319
10Wr	G_SW1	117	117	240	242	187	187	151	151	152	152	311	319
12Wr	G_SW1	117	117	246	246	187	191	151	151	152	156	315	319
17Wr	G_SW1	117	117	246	248	191	195	147	151	156	156	319	319
26Wr	G_SW1	117	117	246	246	191	191	147	151	152	156	319	319
21Wr	G_SW1	113	117	244	246	187	187	147	151	152	152	315	319
8Wr	G_SW1	117	117	246	246	187	195	151	151	152	156	319	319
4Wr	G_SW1	117	117	242	246	187	187	151	151	156	156	315	331
25Wr	G_SW1	113	113	240	246	195	195	151	151	156	156	319	327
30Wr	G_SW1	113	117	246	246	187	195	147	151	152	156	311	319
3Wr	G_SW1	117	117	246	246	187	187	151	151	152	156	311	315
14Wr	G_SW1	117	117	242	242	187	191	147	151	152	156	311	319
20Wr	G_SW1	117	117	242	246	187	187	147	151	152	152	0	0
5Wr	G_SW1	113	117	246	246	187	191	151	151	152	156	311	319
31Wr	G_SW1	113	117	246	246	187	195	147	151	148	156	311	319
9Wr	G_SW1	117	117	244	244	187	187	151	151	148	156	311	315
22Wr	G_SW1	117	117	244	246	195	195	147	151	152	156	311	331
2Wr	G_SW1	117	117	244	246	187	195	151	151	152	156	319	323
18Wr	G_SW1	113	117	246	246	187	195	151	151	148	156	319	323
19Wr	G_SW1	117	119	242	244	187	187	151	151	0	0	311	311
28Wr	G_SW1	113	113	242	246	195	195	151	151	152	152	319	319
7Wr	G_SW1	117	117	242	246	187	195	145	147	152	156	319	319

Number	SITE	Ma8	Ma8	Lut615	Lut615	Lut27	Lut27	Mp059	Mp059	Mf3.2	Mf3.2	Mf4.10	Mf4.10
23Wr	G_SW1	113	117	242	246	187	195	151	151	156	156	307	327
27Wr	G_SW1	117	117	242	246	191	195	147	151	152	156	315	319
224km	G_SW1	113	113	242	246	187	195	147	153	156	160	311	323
346km	G_SW1	117	117	244	246	187	187	151	151	152	156	311	311
309km	H_W1	117	119	240	242	187	187	151	151	152	156	307	327
453km	H_W1	113	117	242	242	187	195	147	151	152	152	311	311
317km	H_W1	113	119	242	242	187	187	147	151	156	156	311	311
321km	H_W1	117	117	240	246	187	187	147	151	152	156	307	327
313km(488)	H_W1	117	117	242	242	187	187	151	151	156	160	311	311
320km	H_W1	113	117	244	244	187	187	147	151	152	156	319	319
491km	H_W1	117	117	242	246	187	187	151	151	156	156	319	319
322km(485)	H_W1	113	119	242	246	187	187	147	151	156	156	319	319
319km	H_W1	117	117	246	246	187	191	147	151	156	156	327	331
489km	H_W1	113	117	242	242	187	191	147	151	156	156	307	315
490km	H_W1	113	117	242	246	187	187	145	151	156	156	311	315
486km	H_W1	113	117	242	244	187	191	147	151	156	156	315	315

CODE	SITE	Mf3.7	Mf3.7	Mf6.5	Mf6.5	Mvi57	Mvi57	Mvi072	Mvi072	Ma2	Ma2	Gg454	GG454
034km	A_NE1	189	193	227	231	106	106	272	274	178	178	128	128
111km	A_NE1	189	189	227	227	106	106	272	272	172	172	128	128
029F	A_NE1	189	189	227	235	106	106	272	272	172	174	128	130
154km	A_NE1	189	189	235	235	106	106	272	272	172	174	128	130
054F	A_NE1	189	189	227	227	106	106	272	272	178	178	128	128
022M	A_NE1	185	189	227	227	106	106	272	272	174	174	130	130
043F	A_NE1	189	189	227	227	106	106	272	272	174	174	128	130
004M	A_NE1	189	189	227	227	106	106	272	272	172	178	128	128
031M	A_NE1	185	189	227	227	106	106	272	272	174	178	128	128
025M(17M)	A_NE1	189	189	227	227	106	106	272	272	172	174	128	128
029M	A_NE1	189	189	227	227	106	106	272	274	172	178	128	130
369km	A_NE1	189	189	227	227	106	106	272	272	174	178	128	130
021F	A_NE1	189	189	227	227	106	106	272	272	174	174	128	128
052F(40F)	A_NE1	185	189	227	235	106	106	272	272	174	178	128	128
025F	A_NE1	189	189	227	227	106	106	272	272	172	174	128	128
024F(289)	A_NE1	185	189	227	227	106	106	272	272	174	178	128	128
035M	A_NE1	185	189	227	235	106	106	272	272	172	174	128	130
198km	A_NE1	189	189	227	227	106	106	272	272	172	174	128	128
026M	A_NE1	189	189	227	227	106	106	272	272	172	174	128	130
032M	A_NE1	189	189	227	235	106	106	272	272	174	178	128	128
110km	A_NE1	189	189	227	227	106	106	272	272	174	178	128	130
006_7M	A_NE1	185	189	231	231	106	106	272	272	174	174	128	130
002F	A_NE1	189	189	227	231	106	106	272	272	174	178	128	128
002km	A_NE1	189	189	227	235	106	106	272	272	172	172	128	128
008F	A_NE1	189	189	227	227	106	106	272	272	172	178	128	128
039F	A_NE1	189	189	227	227	106	106	272	272	174	174	130	130
197km	A_NE1	189	189	227	227	106	106	272	274	172	172	128	128
087km	A_NE1	189	189	227	235	106	106	272	272	172	178	128	128
009M	A_NE1	189	189	227	235	106	106	272	272	172	178	128	128
007M	A_NE1	189	189	227	235	106	106	272	272	172	178	128	128
001M	A_NE1	185	189	227	227	106	106	272	272	172	172	128	128
013km	A_NE1	185	189	227	235	106	106	272	272	172	172	128	128

CODE	SITE	Mf3.7	Mf3.7	Mf6.5	Mf6.5	Mvi57	Mvi57	Mvi072	Mvi072	Ma2	Ma2	Gg454	GG454
031F	A_NE1	189	189	235	235	106	106	272	272	174	178	128	128
057km(5M)	A_NE1	185	189	227	235	106	106	272	272	172	172	128	128
047F	A_NE1	189	189	227	235	106	106	272	272	174	178	128	130
016M	A_NE1	189	189	227	235	106	106	272	272	172	172	128	128
106km	A_NE1	189	189	227	235	106	106	272	272	172	172	128	128
042F	A_NE1	185	185	227	235	106	106	272	272	172	174	128	130
023F	A_NE1	185	189	227	231	106	106	272	272	172	178	128	128
006M	A_NE1	189	189	227	235	106	106	272	272	172	174	128	130
050F	A_NE1	185	189	227	235	106	106	272	272	172	172	128	128
325km	A_NE1	185	189	227	235	106	106	272	274	172	178	128	128
38F	A_NE1	185	189	231	235	106	106	272	272	172	178	128	128
019M	A_NE1	185	185	227	231	106	106	272	272	172	178	128	128
049F	A_NE1	189	189	227	227	106	106	272	272	174	178	128	128
023M	A_NE1	185	189	231	231	106	106	272	272	172	172	128	128
090km	A_NE1	185	185	231	235	106	106	272	272	172	172	128	128
033M	A_NE1	189	189	235	235	106	106	272	272	178	178	128	128
028F	A_NE1	189	189	227	227	106	106	272	274	172	172	128	128
207km	A_NE1	189	189	235	235	106	106	272	272	172	172	128	128
055F	A_NE1	185	189	231	235	106	106	272	272	172	178	128	128
022km	A_NE1	189	189	227	235	106	106	272	272	172	174	128	130
056km	A_NE1	185	189	227	235	106	106	272	272	172	178	128	130
123km	A_NE1	189	189	227	235	106	106	272	272	172	178	128	128
113km	A_NE1	185	193	231	235	106	106	272	272	172	178	128	128
348km	A_NE1	189	189	227	235	106	106	272	272	172	172	128	128
112km	A_NE1	189	193	235	235	106	106	270	272	174	178	128	128
203km	A_NE1	189	189	227	235	106	106	272	272	172	172	128	128
450km	B_NE2	185	189	227	235	106	106	272	272	172	174	128	128
499km	B_NE2	189	189	227	235	106	108	272	272	172	172	128	128
296km	B_NE2	185	189	227	227	106	106	272	272	174	174	128	128
349km	B_NE2	185	189	227	227	106	106	272	272	172	172	128	128
354km	B_NE2	185	185	227	227	106	106	272	274	174	178	128	130
225km	B_NE2	189	189	227	231	106	106	272	274	172	178	128	128

CODE	SITE	Mf3.7	Mf3.7	Mf6.5	Mf6.5	Mvi57	Mvi57	Mvi072	Mvi072	Ma2	Ma2	Gg454	GG454
316km	B_NE2	185	189	227	235	106	106	272	272	172	172	128	128
396km	B_NE2	185	189	227	235	106	106	272	272	172	172	128	128
315km	B_NE2	189	189	227	235	106	106	272	272	172	178	128	128
223km	B_NE2	189	189	227	231	106	106	272	274	172	178	128	128
045F	B_NE2	189	189	231	239	106	106	272	272	178	178	128	128
003km	B_NE2	185	189	235	235	106	106	272	272	172	178	128	128
393km	B_NE2	185	189	227	231	106	106	272	272	178	178	128	128
018M	B_NE2	185	189	231	235	106	106	272	272	172	178	128	128
046F	B_NE2	189	189	227	235	106	106	272	274	172	178	128	128
20F	B_NE2	185	189	227	231	106	106	272	272	174	178	128	128
028M	B_NE2	185	189	235	235	106	106	272	272	174	174	128	130
034M	B_NE2	185	189	227	231	106	106	272	272	174	178	128	128
226km	B_NE2	189	189	227	231	106	106	272	274	172	178	128	128
022F	B_NE2	189	189	227	227	106	106	272	272	178	178	128	128
037F	B_NE2	185	185	227	231	106	106	272	272	172	174	128	128
053F	B_NE2	185	189	227	231	106	106	272	272	174	178	128	128
020M	B_NE2	185	189	227	231	0	0	0	0	174	174	128	128
493km	B_NE2	189	189	227	227	106	106	272	272	172	178	128	128
536km	B_NE2	185	193	227	227	106	106	272	272	172	178	128	128
021M(451)	B_NE2	185	185	227	227	106	106	272	272	172	172	128	128
051F	B_NE2	185	189	227	235	106	106	272	272	172	172	128	128
044F	B_NE2	185	189	227	235	106	106	272	272	172	174	130	130
027M	B_NE2	189	189	227	227	106	106	272	272	172	172	128	128
239km	B_NE2	189	189	227	227	106	108	272	272	174	178	128	130
368km	B_NE2	185	189	235	235	106	106	274	274	174	178	128	128
048F	B_NE2	185	189	227	235	106	106	272	272	172	172	128	128
147km	B_NE2	189	189	227	231	106	106	272	274	172	174	128	128
034F	B_NE2	185	185	227	235	106	106	272	272	172	178	128	128
116km	B_NE2	185	185	227	227	106	106	272	272	174	174	128	130
210km	B_NE2	185	189	227	235	106	106	272	272	174	178	128	128
405km	B_NE2	189	189	227	231	106	106	272	272	172	178	128	128
323km	B_NE2	189	189	227	235	106	106	272	272	174	174	128	128

CODE	SITE	Mf3.7	Mf3.7	Mf6.5	Mf6.5	Mvi57	Mvi57	Mvi072	Mvi072	Ma2	Ma2	Gg454	GG454
476km	B_NE2	189	189	227	227	106	106	272	274	174	174	128	128
297km	B_NE2	185	185	227	235	106	106	272	272	172	174	128	128
324km	B_NE2	185	189	227	235	106	106	270	272	174	178	128	128
241km	B_NE2	185	189	227	227	106	106	270	272	178	180	0	0
217km	B_NE2	189	189	227	235	106	106	272	274	172	178	128	128
138km	B_NE2	189	189	227	231	106	106	272	272	172	178	128	128
455km	B_NE2	185	189	227	231	106	108	272	278	174	178	128	128
211km	B_NE2	189	189	227	235	106	108	272	272	174	178	128	130
222km	B_NE2	185	189	227	227	106	106	270	272	174	178	128	128
108km	B_NE2	185	189	227	231	106	106	272	272	174	178	128	128
085km	B_NE2	189	189	227	227	106	106	272	272	174	178	128	128
127km	B_NE2	185	189	227	227	106	108	272	272	174	178	128	128
044km	B_NE2	185	185	235	235	106	106	272	272	172	172	128	128
282km	B_NE2	185	193	227	231	106	106	272	272	174	174	128	128
403km	B_NE2	189	189	235	235	106	106	272	272	172	178	128	128
365km	B_NE2	189	189	227	227	106	106	274	274	174	174	128	130
371km	B_NE2	185	189	227	235	106	106	274	274	172	174	128	128
137km	B_NE2	185	189	227	231	106	106	272	274	174	178	128	128
299km	B_NE2	185	189	235	235	106	106	272	272	174	178	128	128
362-2km	B_NE2	189	189	227	235	106	106	272	272	172	174	128	128
308km	B_NE2	185	189	227	235	106	106	272	274	172	178	128	128
140km	B_NE2	189	189	227	231	106	106	272	272	172	172	128	128
238km	B_NE2	189	189	231	235	106	108	272	272	172	174	128	128
402km	B_NE2	185	185	227	235	106	108	272	274	174	174	128	128
397km	B_NE2	185	189	227	235	106	106	274	274	174	178	128	130
270km	B_NE2	189	193	227	235	106	106	272	272	174	178	128	128
276km	B_NE2	185	189	227	235	106	106	272	274	178	178	128	128
474km	B_NE2	185	189	227	227	106	106	272	272	172	174	128	128
220_7km	B_NE2	189	189	231	235	106	106	272	272	172	174	128	128
220_6km	B_NE2	189	189	231	235	106	106	272	272	174	178	128	128
220_2km	B_NE2	189	189	227	235	106	106	272	272	174	174	128	128
220_4km	B_NE2	189	189	235	235	106	106	272	272	172	178	128	128

CODE	SITE	Mf3.7	Mf3.7	Mf6.5	Mf6.5	Mvi57	Mvi57	Mvi072	Mvi072	Ma2	Ma2	Gg454	GG454
220_3km	B_NE2	185	189	227	227	106	108	272	272	174	178	128	130
220_1km	B_NE2	189	189	231	235	106	106	272	272	172	178	128	128
267km	B_NE2	185	193	227	235	106	106	272	272	174	178	128	128
372km	B_NE2	189	189	235	235	106	106	272	272	174	178	128	128
287km	B_NE2	185	189	235	235	106	106	272	272	178	178	128	130
286km	B_NE2	189	189	227	227	106	106	272	274	174	174	128	130
284km	B_NE2	185	185	235	235	106	106	272	274	174	178	128	130
283km	B_NE2	185	189	227	235	106	106	272	274	174	174	128	130
281km	B_NE2	189	189	227	235	106	106	272	272	172	174	128	130
292km	B_NE2	189	189	227	235	106	106	272	272	172	178	128	128
295km	B_NE2	185	189	227	239	106	106	272	274	174	178	128	128
234km	B_NE2	189	189	227	235	106	106	272	274	178	178	128	128
377km	B_NE2	189	189	227	235	106	106	272	274	174	178	128	128
437km	B_NE2	185	189	227	235	106	106	274	274	172	174	128	128
229km	B_NE2	189	189	235	235	106	106	272	272	172	172	128	128
269km	B_NE2	193	193	0	0	106	106	272	272	178	178	128	128
357km	B_NE2	189	189	227	231	106	108	274	274	172	178	128	128
358km	B_NE2	185	189	227	227	106	106	272	272	178	178	128	128
237km	B_NE2	189	189	227	235	106	106	272	272	174	178	128	128
376km	B_NE2	185	189	227	235	106	106	272	272	174	174	128	130
503km	B_NE2	189	189	227	227	106	106	272	272	172	178	128	128
367km	B_NE2	189	189	227	235	106	108	272	272	172	178	128	128
404km	B_NE2	189	189	227	235	106	108	272	274	174	174	128	128
406km	B_NE2	189	193	235	235	106	106	272	272	174	178	128	130
208km	B_NE2	189	189	227	239	106	106	272	274	178	178	128	130
522km	C_NE3	185	185	227	235	106	106	272	274	172	174	128	130
446km	C_NE3	185	189	227	231	106	108	272	272	174	178	128	130
543km	C_NE3	185	189	227	227	106	108	272	274	174	178	128	128
353_1km(PC	C_NE3	189	189	227	235	106	106	272	274	174	178	128	128
527km	C_NE3	185	189	227	227	106	106	272	272	174	174	130	130
448km	C_NE3	185	189	235	235	106	106	272	272	178	178	128	128
274km	C_NE3	185	185	227	231	106	108	274	274	178	178	128	128

CODE	SITE	Mf3.7	Mf3.7	Mf6.5	Mf6.5	Mvi57	Mvi57	Mvi072	Mvi072	Ma2	Ma2	Gg454	GG454
311km	C_NE3	185	193	231	235	106	106	272	274	172	174	128	128
248km	C_NE3	185	189	227	235	106	106	272	274	172	178	128	128
244km	C_NE3	189	189	227	227	106	108	272	272	174	178	128	128
443km	C_NE3	193	193	227	235	106	106	272	272	172	174	128	128
246km	C_NE3	189	193	227	235	106	106	272	274	174	178	128	128
440km	C_NE3	185	185	227	235	106	106	272	272	178	178	128	128
442km	C_NE3	185	189	227	227	106	108	272	272	174	178	128	128
441km	C_NE3	189	189	227	235	106	106	272	274	172	174	128	128
179km	C_NE3	185	189	231	235	106	106	272	272	174	178	128	128
245-akm	C_NE3	185	189	227	235	106	106	272	272	172	178	128	128
245km	C_NE3	189	189	227	227	106	106	272	272	172	174	130	130
232_1km	C_NE3	185	189	227	235	106	106	274	274	178	178	128	128
232_2km	C_NE3	185	193	227	227	106	106	272	274	174	178	128	128
436km	C_NE3	185	189	231	235	106	106	272	274	178	178	128	130
249km	C_NE3	189	193	235	235	106	106	272	272	174	174	128	130
243km	C_NE3	185	189	227	231	106	106	272	274	178	180	128	128
247km	C_NE3	189	189	231	231	106	108	272	272	174	178	128	128
449km	C_NE3	185	189	227	235	106	106	272	274	172	178	128	128
086km	D_CE1	189	189	231	235	106	106	272	272	174	174	128	128
391km	D_CE1	189	189	227	227	106	106	274	274	178	178	128	128
169km	D_CE1	185	189	227	231	106	106	272	274	174	178	128	128
326km	D_CE1	189	193	227	235	106	106	272	272	174	178	128	128
300km	D_CE1	189	193	231	235	106	106	272	274	172	174	128	130
411km	D_CE1	189	189	227	235	106	108	272	274	172	174	128	128
115km(PCR1	D_CE1	185	189	231	235	106	106	272	274	174	178	128	128
395km	D_CE1	189	189	227	227	106	106	272	272	174	174	128	128
305km	D_CE1	185	185	227	235	106	108	272	272	178	178	128	128
167km	D_CE1	185	189	227	227	106	106	272	272	178	178	128	128
519km	D_CE1	185	189	227	235	106	106	272	272	178	178	128	128
475km	D_CE1	189	189	227	235	106	106	272	274	178	178	128	130
242km	D_CE1	189	189	227	231	106	108	272	272	172	178	128	128
228km	D_CE1	189	189	227	227	106	106	272	274	178	178	128	130

CODE	SITE	Mf3.7	Mf3.7	Mf6.5	Mf6.5	Mvi57	Mvi57	Mvi072	Mvi072	Ma2	Ma2	Gg454	GG454
212km	D_CE1	189	189	235	235	106	106	272	272	174	178	128	130
107km	E_CE2	185	189	243	243	106	108	274	274	172	172	128	128
484km	E_CE2	189	189	227	227	106	108	272	272	172	178	128	128
480km	E_CE2	189	189	235	235	108	108	274	274	172	172	128	128
481km	E_CE2	189	189	231	235	106	106	272	272	172	178	128	128
398km	E_CE2	189	189	231	231	108	108	272	274	174	174	130	130
252km	E_CE2	185	189	227	235	106	106	272	272	172	174	128	128
253km	E_CE2	189	189	227	235	106	106	272	272	174	174	128	128
236km	E_CE2	189	189	227	235	106	106	272	272	172	178	128	128
260km	E_CE2	185	189	235	235	106	106	272	274	178	178	128	128
250km	E_CE2	185	185	227	231	106	106	272	272	174	178	128	128
262km	E_CE2	189	189	227	227	106	106	272	272	174	178	128	128
265km	E_CE2	185	189	227	227	106	106	272	274	172	172	128	128
168km	E_CE2	189	189	227	235	106	106	272	274	174	178	128	128
258km	E_CE2	185	189	235	235	106	106	272	272	172	172	128	128
255km	E_CE2	185	189	227	231	106	108	274	274	178	178	128	130
256km	E_CE2	189	193	235	235	106	108	272	272	172	174	128	128
264km	E_CE2	185	185	227	235	106	106	272	274	178	178	128	130
251km	E_CE2	185	185	235	235	106	106	272	274	172	178	128	128
261km	E_CE2	185	189	235	235	106	106	272	274	172	178	128	128
257km	E_CE2	189	189	227	231	106	106	272	274	174	178	128	128
254km	E_CE2	185	189	227	235	106	106	272	272	174	178	128	128
263km	E_CE2	185	189	227	235	106	106	272	274	178	178	128	128
333km	F_SE1	189	189	227	227	106	106	272	274	174	178	128	128
29Wr	F_SE1	189	189	227	235	108	108	272	274	174	178	128	130
386km	F_SE1	185	189	227	227	102	106	274	274	174	174	128	128
388km	F_SE1	185	189	227	235	106	106	272	274	174	174	128	128
383km	F_SE1	189	189	235	235	106	106	272	274	174	178	128	128
381km	F_SE1	185	185	235	235	108	108	272	274	174	174	126	128
385km	F_SE1	185	185	235	235	102	106	272	274	174	174	128	128
382km	F_SE1	185	189	227	227	106	106	272	274	174	178	128	130
380km	F_SE1	185	185	227	239	106	106	274	274	172	174	128	128

CODE	SITE	Mf3.7	Mf3.7	Mf6.5	Mf6.5	Mvi57	Mvi57	Mvi072	Mvi072	Ma2	Ma2	Gg454	GG454
384km	F_SE1	185	189	227	235	106	106	272	274	174	174	128	128
390km	F_SE1	189	193	227	227	102	108	274	274	178	178	128	128
387km	F_SE1	189	189	227	227	106	106	274	276	172	178	128	128
389km	F_SE1	189	193	231	235	102	106	272	274	172	174	128	128
378km	F_SE1	185	185	231	235	106	106	272	274	178	178	128	128
1Wr	G_SW1	185	189	227	231	106	108	274	274	172	172	128	130
16Wr	G_SW1	189	189	227	227	106	106	274	274	178	178	128	128
11Wr	G_SW1	189	189	235	235	106	106	272	274	172	172	128	128
6Wr	G_SW1	185	189	227	235	106	106	272	274	172	172	128	128
15Wr	G_SW1	185	189	227	235	106	108	272	274	174	178	128	130
13Wr	G_SW1	189	189	227	231	106	106	272	274	172	174	128	128
10Wr	G_SW1	189	189	227	235	106	106	272	272	172	178	128	128
12Wr	G_SW1	185	189	227	227	106	106	272	274	172	178	128	128
17Wr	G_SW1	185	189	227	235	106	106	272	272	172	174	128	128
26Wr	G_SW1	189	189	227	227	106	108	272	274	174	174	128	130
21Wr	G_SW1	189	193	227	235	106	106	274	274	178	178	128	128
8Wr	G_SW1	189	189	227	227	106	108	274	274	172	178	128	128
4Wr	G_SW1	189	193	227	235	106	108	272	274	174	178	128	128
25Wr	G_SW1	189	189	0	0	106	106	272	274	178	178	128	130
30Wr	G_SW1	189	189	227	227	106	106	272	272	172	178	128	130
3Wr	G_SW1	189	189	231	235	108	108	274	276	174	174	128	128
14Wr	G_SW1	189	193	235	235	106	108	272	274	172	174	128	128
20Wr	G_SW1	185	189	0	0	106	108	272	274	172	174	128	128
5Wr	G_SW1	189	189	227	227	106	106	272	272	178	178	128	128
31Wr	G_SW1	189	189	227	231	106	108	272	272	172	174	128	128
9Wr	G_SW1	185	189	235	235	106	106	272	272	174	174	128	128
22Wr	G_SW1	185	189	235	235	106	106	272	274	172	174	128	128
2Wr	G_SW1	189	189	227	235	106	106	274	276	172	174	128	128
18Wr	G_SW1	189	189	227	227	106	108	274	274	174	174	128	130
19Wr	G_SW1	0	0	235	235	106	106	272	272	172	178	128	128
28Wr	G_SW1	189	189	227	227	106	106	272	272	174	174	128	128
7Wr	G_SW1	185	189	235	235	106	106	272	276	172	174	128	128

CODE	SITE	Mf3.7	Mf3.7	Mf6.5	Mf6.5	Mvi57	Mvi57	Mvi072	Mvi072	Ma2	Ma2	Gg454	GG454
23Wr	G_SW1	185	185	227	227	106	106	272	274	166	172	128	128
27Wr	G_SW1	185	189	231	235	106	106	272	276	172	174	128	128
224km	G_SW1	185	193	227	235	106	106	272	274	172	178	128	130
346km	G_SW1	185	185	231	235	106	108	272	276	172	172	128	128
309km	H_W1	189	189	227	235	106	106	272	272	174	178	128	128
453km	H_W1	189	189	231	235	106	106	274	274	174	178	128	130
317km	H_W1	189	193	227	231	106	106	270	272	172	178	128	128
321km	H_W1	189	189	227	235	106	106	272	272	172	178	128	128
313km(488)	H_W1	189	189	231	235	106	106	272	272	174	178	128	128
320km	H_W1	189	193	227	227	106	108	272	272	172	178	128	128
491km	H_W1	185	189	227	227	106	106	272	272	172	174	128	130
322km(485)	H_W1	189	189	231	235	106	106	272	272	172	172	128	130
319km	H_W1	189	189	235	235	106	106	272	272	172	178	128	130
489km	H_W1	189	189	231	235	106	106	274	274	172	178	130	130
490km	H_W1	189	189	227	231	106	106	272	274	174	178	128	130
486km	H_W1	189	189	227	227	106	108	272	274	174	178	128	128

CODE	SITE	Mel1	Mel1	Mer041	Mer041	Ma1	Ma1	Mer43	Mer43	Mer15	Mer15	Mf4.17	Mf4.17
034km	A_NE1	270	270	159	159	202	206	141	141	183	189	203	215
111km	A_NE1	264	270	159	159	202	204	141	143	183	189	203	219
029F	A_NE1	270	270	159	159	202	204	141	141	189	189	219	219
154km	A_NE1	268	268	159	159	204	204	141	141	189	189	215	219
054F	A_NE1	270	270	159	159	204	204	141	143	183	183	211	227
022M	A_NE1	264	268	159	171	206	206	141	143	183	189	215	215
043F	A_NE1	264	268	159	171	206	206	141	143	189	189	215	215
004M	A_NE1	268	270	159	159	204	204	141	141	183	189	219	219
031M	A_NE1	264	268	159	159	202	204	141	143	183	189	215	215
025M(17M)	A_NE1	268	268	159	159	202	206	141	141	183	189	211	219
029M	A_NE1	270	270	159	159	204	206	141	141	185	189	215	223
369km	A_NE1	264	264	159	159	204	204	141	141	189	189	215	215
021F	A_NE1	260	268	159	159	206	206	141	141	183	189	215	219
052F(40F)	A_NE1	260	268	159	159	202	206	141	141	183	183	211	215
025F	A_NE1	270	270	159	159	204	206	141	143	185	189	211	215
024F(289)	A_NE1	260	268	159	159	202	206	141	141	183	189	215	219
035M	A_NE1	264	270	159	171	202	204	141	143	183	189	215	215
198km	A_NE1	268	270	159	159	202	206	141	143	183	189	211	211
026M	A_NE1	268	270	159	159	202	204	141	143	183	189	203	219
032M	A_NE1	268	268	159	159	202	204	141	141	183	189	215	219
110km	A_NE1	260	268	159	159	206	206	141	141	183	189	211	219
006_7M	A_NE1	268	270	159	159	206	206	141	141	183	183	215	219
002F	A_NE1	268	270	159	159	206	206	141	141	183	183	215	219
002km	A_NE1	268	270	159	159	202	204	141	143	183	189	211	215
008F	A_NE1	268	268	159	165	204	204	141	141	183	189	215	219
039F	A_NE1	264	270	159	159	204	206	141	143	183	189	215	215
197km	A_NE1	268	270	159	159	206	206	141	141	183	189	211	215
087km	A_NE1	268	270	159	159	204	204	141	141	183	189	215	219
009M	A_NE1	264	268	159	159	204	204	141	141	183	189	215	219
007M	A_NE1	268	268	159	165	204	204	141	141	189	189	215	219
001M	A_NE1	270	270	159	159	202	202	141	141	183	183	219	219
013km	A_NE1	270	270	159	159	204	204	141	141	189	189	215	219

CODE	SITE	Mel1	Mel1	Mer041	Mer041	Ma1	Ma1	Mer43	Mer43	Mer15	Mer15	Mf4.17	Mf4.17
031F	A_NE1	268	270	159	159	202	206	141	143	183	183	211	219
057km(5M)	A_NE1	270	270	159	159	202	204	141	141	183	189	211	219
047F	A_NE1	264	270	159	159	202	204	141	141	189	189	215	215
016M	A_NE1	268	270	159	165	204	204	141	141	189	189	211	219
106km	A_NE1	270	270	159	159	204	204	141	143	183	189	215	219
042F	A_NE1	270	270	159	159	206	206	141	141	183	183	215	215
023F	A_NE1	260	270	159	159	204	206	141	141	183	183	211	215
006M	A_NE1	264	270	159	159	204	204	141	141	183	189	215	219
050F	A_NE1	264	270	159	159	204	204	141	141	183	189	215	219
325km	A_NE1	268	270	159	171	202	206	141	141	183	183	215	219
38F	A_NE1	270	270	159	171	206	206	141	141	183	189	211	219
019M	A_NE1	270	270	159	159	204	206	141	143	183	183	211	219
049F	A_NE1	268	270	159	159	202	204	141	143	183	183	211	211
023M	A_NE1	270	270	159	171	206	206	141	143	183	183	211	215
090km	A_NE1	266	270	159	171	206	206	141	141	183	183	211	215
033M	A_NE1	268	270	159	159	204	204	141	141	189	189	219	219
028F	A_NE1	270	270	165	171	204	204	141	143	189	189	215	215
207km	A_NE1	264	270	159	159	204	204	141	141	189	189	219	219
055F	A_NE1	270	270	159	159	204	204	141	141	183	183	215	219
022km	A_NE1	260	270	159	159	202	204	141	143	183	183	215	215
056km	A_NE1	260	270	159	159	202	204	141	143	183	189	203	219
123km	A_NE1	270	270	159	159	204	204	141	141	183	189	211	219
113km	A_NE1	260	260	159	171	206	206	141	141	183	189	219	219
348km	A_NE1	264	268	159	159	202	204	141	141	189	189	215	215
112km	A_NE1	260	260	159	171	202	202	141	141	183	189	219	231
203km	A_NE1	270	270	159	159	202	206	141	141	183	189	211	219
450km	B_NE2	266	268	159	159	206	206	141	141	183	183	219	219
499km	B_NE2	270	270	159	159	202	204	141	143	183	189	219	223
296km	B_NE2	264	270	159	171	202	204	141	143	189	189	215	215
349km	B_NE2	270	270	167	171	202	206	141	143	183	189	215	215
354km	B_NE2	260	270	159	159	206	206	141	141	183	183	203	211
225km	B_NE2	260	260	159	159	202	202	141	141	183	183	215	219

CODE	SITE	Mel1	Mel1	Mer041	Mer041	Ma1	Ma1	Mer43	Mer43	Mer15	Mer15	Mf4.17	Mf4.17
316km	B_NE2	270	270	159	171	204	206	141	141	183	183	215	219
396km	B_NE2	270	270	159	167	202	206	141	141	183	183	211	215
315km	B_NE2	266	270	159	159	206	206	141	141	183	189	215	223
223km	B_NE2	270	270	159	159	202	206	141	143	183	185	211	215
045F	B_NE2	270	270	159	171	202	204	141	141	183	189	203	203
003km	B_NE2	268	270	159	167	206	206	141	141	183	183	215	215
393km	B_NE2	260	260	159	167	206	206	141	141	183	183	215	215
018M	B_NE2	260	270	159	159	202	206	141	141	183	183	203	215
046F	B_NE2	260	270	159	165	204	204	141	143	183	189	211	211
20F	B_NE2	260	260	159	159	202	206	141	143	183	183	203	215
028M	B_NE2	260	268	159	167	202	202	141	141	183	185	203	223
034M	B_NE2	260	270	159	171	204	204	141	141	189	189	203	215
226km	B_NE2	260	270	159	171	202	202	141	141	183	183	211	211
022F	B_NE2	260	260	171	171	202	204	143	143	183	183	215	215
037F	B_NE2	266	270	159	159	206	206	141	141	183	189	211	231
053F	B_NE2	260	266	159	159	202	202	141	141	183	189	215	231
020M	B_NE2	260	270	159	167	206	206	141	141	183	189	219	231
493km	B_NE2	260	270	159	159	206	206	141	141	183	183	207	215
536km	B_NE2	260	268	159	167	204	204	141	141	183	189	203	223
021M(451)	B_NE2	260	264	159	167	202	206	141	143	183	183	203	215
051F	B_NE2	264	270	159	167	202	206	143	143	183	185	215	223
044F	B_NE2	260	270	159	159	206	206	141	143	183	183	215	215
027M	B_NE2	260	264	159	159	206	206	141	141	183	185	215	223
239km	B_NE2	270	270	159	167	204	204	141	143	183	183	215	219
368km	B_NE2	260	270	159	159	204	204	141	141	183	183	215	219
048F	B_NE2	264	270	159	167	206	206	141	141	183	185	215	215
147km	B_NE2	260	268	159	159	206	206	141	141	183	183	211	215
034F	B_NE2	260	270	159	167	202	206	141	141	183	185	215	223
116km	B_NE2	260	270	159	159	206	206	141	141	183	183	211	235
210km	B_NE2	268	268	171	171	202	202	141	141	183	189	215	219
405km	B_NE2	260	268	159	159	204	206	141	143	183	189	215	215
323km	B_NE2	270	270	159	159	202	204	141	143	183	183	211	215

CODE	SITE	Mel1	Mel1	Mer041	Mer041	Ma1	Ma1	Mer43	Mer43	Mer15	Mer15	Mf4.17	Mf4.17
476km	B_NE2	260	270	159	171	202	202	141	141	183	183	215	231
297km	B_NE2	260	260	159	171	202	202	141	141	183	183	211	227
324km	B_NE2	260	270	159	171	202	206	143	143	183	183	215	227
241km	B_NE2	260	260	159	159	202	206	141	141	183	183	211	211
217km	B_NE2	268	270	159	159	202	204	141	141	183	189	203	211
138km	B_NE2	268	268	159	167	204	204	141	141	183	183	203	231
455km	B_NE2	268	270	159	167	202	206	141	143	183	183	219	231
211km	B_NE2	270	270	159	159	202	206	141	141	183	185	223	231
222km	B_NE2	270	270	159	159	206	206	141	143	183	185	203	227
108km	B_NE2	268	270	159	167	206	206	141	143	183	189	215	219
085km	B_NE2	260	270	159	171	202	202	141	141	183	185	203	219
127km	B_NE2	264	270	159	159	202	202	141	141	183	183	203	203
044km	B_NE2	270	270	159	159	206	206	141	143	183	183	211	215
282km	B_NE2	268	270	159	165	206	206	141	141	183	183	215	215
403km	B_NE2	268	268	159	159	202	206	141	141	183	189	215	215
365km	B_NE2	260	270	167	167	202	206	141	141	183	183	215	219
371km	B_NE2	260	270	159	171	202	206	141	141	183	183	215	235
137km	B_NE2	268	270	159	171	202	204	141	141	183	183	215	215
299km	B_NE2	270	270	159	159	206	206	141	141	183	185	215	215
362-2km	B_NE2	260	270	159	171	202	206	141	141	183	189	215	219
308km	B_NE2	268	270	159	171	202	202	141	143	183	183	211	215
140km	B_NE2	270	270	159	159	206	206	141	143	183	189	219	227
238km	B_NE2	270	270	159	159	206	206	141	143	183	189	215	219
402km	B_NE2	270	270	159	171	202	202	141	141	183	183	215	215
397km	B_NE2	268	270	159	159	206	206	141	143	189	189	211	219
270km	B_NE2	270	270	159	171	206	206	141	141	183	189	219	235
276km	B_NE2	270	270	159	159	206	206	141	141	183	185	223	231
474km	B_NE2	268	270	159	159	202	206	141	143	183	189	211	231
220_7km	B_NE2	268	270	167	167	202	206	143	143	183	189	231	231
220_6km	B_NE2	270	270	159	165	202	206	141	141	183	189	211	215
220_2km	B_NE2	268	270	159	167	202	206	141	141	183	185	211	235
220_4km	B_NE2	270	270	159	167	202	204	141	143	189	189	215	231

CODE	SITE	Mel1	Mel1	Mer041	Mer041	Ma1	Ma1	Mer43	Mer43	Mer15	Mer15	Mf4.17	Mf4.17
220_3km	B_NE2	270	270	165	167	202	206	141	141	183	183	215	223
220_1km	B_NE2	268	270	167	167	206	206	141	143	185	189	211	231
267km	B_NE2	270	270	159	159	206	206	141	143	183	183	215	219
372km	B_NE2	266	268	159	171	202	206	141	141	183	189	211	219
287km	B_NE2	260	270	159	167	202	206	141	141	185	189	211	215
286km	B_NE2	260	270	159	167	202	202	141	143	183	189	203	215
284km	B_NE2	266	268	159	167	202	206	141	143	183	183	211	215
283km	B_NE2	268	268	159	167	202	206	141	141	183	189	203	211
281km	B_NE2	268	268	159	167	206	206	141	141	183	183	215	215
292km	B_NE2	260	270	159	159	202	206	141	141	183	183	203	215
295km	B_NE2	270	270	167	171	202	204	141	143	183	189	219	219
234km	B_NE2	270	270	171	171	202	204	141	141	183	183	215	215
377km	B_NE2	260	270	159	159	202	202	141	141	183	189	211	211
437km	B_NE2	260	260	159	165	202	206	141	143	183	185	203	223
229km	B_NE2	260	270	165	167	202	206	141	141	183	183	215	223
269km	B_NE2	270	270	0	0	0	0	141	143	183	185	211	231
357km	B_NE2	268	270	159	163	202	202	141	141	183	183	215	219
358km	B_NE2	270	270	159	171	206	206	141	141	183	183	211	211
237km	B_NE2	268	270	159	159	202	206	141	141	183	183	207	215
376km	B_NE2	268	270	159	159	206	206	141	141	183	189	203	219
503km	B_NE2	268	270	159	159	206	206	141	143	183	185	203	215
367km	B_NE2	268	270	159	171	202	206	0	0	0	0	0	0
404km	B_NE2	266	270	159	167	206	206	141	141	183	183	215	219
406km	B_NE2	260	270	159	171	202	202	141	141	183	183	211	211
208km	B_NE2	270	270	159	159	202	202	141	143	183	189	211	219
522km	C_NE3	270	270	159	165	202	202	141	143	183	189	211	215
446km	C_NE3	270	270	159	167	202	202	141	143	183	189	215	215
543km	C_NE3	260	268	167	171	206	206	141	143	185	189	203	219
353_1km(PC	C_NE3	268	270	165	171	202	202	141	141	183	183	215	219
527km	C_NE3	268	268	167	167	206	206	141	143	183	185	215	215
448km	C_NE3	268	270	165	167	206	206	141	141	183	185	203	215
274km	C_NE3	268	270	159	159	202	202	141	141	183	185	215	215

CODE	SITE	Mel1	Mel1	Mer041	Mer041	Ma1	Ma1	Mer43	Mer43	Mer15	Mer15	Mf4.17	Mf4.17
311km	C_NE3	266	270	167	171	200	200	141	145	183	183	215	219
248km	C_NE3	268	270	159	159	202	202	141	143	183	183	203	211
244km	C_NE3	266	270	159	171	206	206	141	141	189	189	215	219
443km	C_NE3	266	268	159	165	202	204	141	141	183	183	215	215
246km	C_NE3	268	270	159	159	206	206	141	141	183	189	215	215
440km	C_NE3	268	270	159	167	202	206	141	141	183	183	211	219
442km	C_NE3	260	268	159	159	206	206	141	141	183	183	219	231
441km	C_NE3	268	270	159	167	202	206	141	141	183	183	203	215
179km	C_NE3	270	270	159	171	202	202	141	141	185	189	215	219
245-akm	C_NE3	268	270	165	171	202	202	141	141	183	189	215	231
245km	C_NE3	268	270	167	171	206	206	141	141	189	189	215	215
232_1km	C_NE3	260	270	159	159	202	202	141	141	183	183	203	215
232_2km	C_NE3	268	270	159	171	206	206	141	141	183	185	211	215
436km	C_NE3	260	268	159	165	206	206	141	141	189	189	223	223
249km	C_NE3	268	270	159	171	202	206	141	141	189	189	215	215
243km	C_NE3	260	270	159	161	202	202	141	141	183	183	215	219
247km	C_NE3	268	270	159	171	206	206	141	141	189	189	215	215
449km	C_NE3	268	270	159	159	206	206	141	141	183	189	211	215
086km	D_CE1	266	270	159	171	202	202	141	141	189	189	211	215
391km	D_CE1	268	270	159	171	202	206	141	143	183	183	207	211
169km	D_CE1	260	268	159	167	206	206	141	141	183	189	211	219
326km	D_CE1	266	268	159	171	206	206	141	143	183	189	207	211
300km	D_CE1	270	270	159	167	206	206	141	141	183	183	203	215
411km	D_CE1	270	270	159	159	206	206	141	141	183	185	215	239
115km(PCR1	D_CE1	268	268	159	171	202	202	141	143	183	183	215	219
395km	D_CE1	260	270	159	171	202	206	141	141	183	183	211	215
305km	D_CE1	268	270	159	165	202	204	141	141	181	183	203	215
167km	D_CE1	260	270	159	167	202	202	141	143	189	189	207	215
519km	D_CE1	260	270	159	167	204	206	141	141	183	183	203	215
475km	D_CE1	260	270	159	159	204	206	141	141	183	189	215	215
242km	D_CE1	268	270	159	159	206	206	141	141	183	189	211	215
228km	D_CE1	266	270	159	171	202	202	141	141	183	183	203	215

CODE	SITE	Mel1	Mel1	Mer041	Mer041	Ma1	Ma1	Mer43	Mer43	Mer15	Mer15	Mf4.17	Mf4.17
212km	D_CE1	270	270	159	159	202	202	141	141	183	185	211	227
107km	E_CE2	260	268	159	159	200	202	141	141	183	183	203	207
484km	E_CE2	268	268	159	159	202	202	141	141	183	183	219	219
480km	E_CE2	268	270	171	171	206	206	141	141	183	183	215	215
481km	E_CE2	268	268	159	165	206	206	141	141	183	183	215	215
398km	E_CE2	268	270	171	171	202	202	143	143	189	189	215	231
252km	E_CE2	268	270	159	159	202	202	141	143	185	189	211	219
253km	E_CE2	270	270	159	171	206	206	141	141	183	183	215	219
236km	E_CE2	270	270	167	167	202	204	141	141	183	183	211	219
260km	E_CE2	266	268	159	159	206	206	141	141	183	183	211	219
250km	E_CE2	260	260	159	161	202	202	141	143	185	189	215	215
262km	E_CE2	260	270	159	171	206	206	143	143	183	183	207	215
265km	E_CE2	260	270	159	159	202	206	141	141	183	183	211	219
168km	E_CE2	260	270	159	159	200	200	141	141	189	189	215	219
258km	E_CE2	270	270	171	171	206	206	141	141	183	183	207	207
255km	E_CE2	268	268	159	159	202	206	141	141	183	183	227	227
256km	E_CE2	270	270	159	159	206	206	141	141	183	189	219	219
264km	E_CE2	268	268	159	159	202	206	141	143	183	189	215	215
251km	E_CE2	268	270	159	171	200	206	141	141	183	183	215	215
261km	E_CE2	260	268	159	159	202	202	141	141	183	183	211	215
257km	E_CE2	268	270	159	161	202	202	141	141	183	183	211	219
254km	E_CE2	260	270	159	171	202	202	141	141	183	189	215	215
263km	E_CE2	270	270	159	159	202	206	141	141	183	185	215	219
333km	F_SE1	268	270	159	165	202	204	141	141	189	189	215	223
29Wr	F_SE1	260	268	167	171	202	206	141	141	183	183	215	219
386km	F_SE1	270	270	159	159	200	200	141	141	189	189	203	203
388km	F_SE1	270	270	159	171	200	206	141	141	183	183	219	219
383km	F_SE1	268	270	159	159	202	206	141	141	185	189	211	223
381km	F_SE1	266	270	159	159	200	202	141	141	183	185	207	215
385km	F_SE1	268	270	159	159	200	202	141	141	185	189	215	215
382km	F_SE1	266	268	159	159	202	206	141	141	183	183	215	219
380km	F_SE1	268	270	159	171	200	206	141	143	185	189	215	215

CODE	SITE	Mel1	Mel1	Mer041	Mer041	Ma1	Ma1	Mer43	Mer43	Mer15	Mer15	Mf4.17	Mf4.17
384km	F_SE1	266	268	159	171	206	206	141	145	183	183	215	219
390km	F_SE1	268	270	159	165	200	202	141	141	185	189	215	219
387km	F_SE1	270	270	159	171	200	206	141	143	183	183	203	215
389km	F_SE1	260	268	159	159	202	206	141	141	183	183	211	215
378km	F_SE1	260	272	159	165	202	206	141	143	183	189	215	219
1Wr	G_SW1	270	270	159	159	202	204	141	141	183	183	215	219
16Wr	G_SW1	268	268	159	159	202	202	141	141	183	189	211	219
11Wr	G_SW1	260	268	171	171	206	206	141	141	183	183	211	219
6Wr	G_SW1	266	268	159	159	200	206	141	141	183	183	211	219
15Wr	G_SW1	260	270	159	159	202	206	141	141	183	189	207	211
13Wr	G_SW1	260	270	159	159	202	206	141	141	183	185	215	219
10Wr	G_SW1	268	270	159	159	200	206	141	141	183	183	219	219
12Wr	G_SW1	260	270	159	159	202	206	141	141	183	183	211	211
17Wr	G_SW1	260	266	159	159	202	204	141	141	183	189	211	215
26Wr	G_SW1	266	270	159	159	202	206	141	141	183	189	207	219
21Wr	G_SW1	270	270	159	159	202	202	141	141	183	189	207	211
8Wr	G_SW1	268	270	159	159	202	206	141	141	183	183	211	219
4Wr	G_SW1	270	270	167	171	202	204	141	141	183	189	215	223
25Wr	G_SW1	260	268	159	159	202	202	141	143	183	183	219	223
30Wr	G_SW1	270	270	159	159	202	206	141	141	183	189	219	219
3Wr	G_SW1	268	270	159	171	202	206	141	141	189	189	215	215
14Wr	G_SW1	260	270	159	159	202	206	141	141	189	189	219	227
20Wr	G_SW1	264	270	159	171	202	202	143	143	183	185	207	211
5Wr	G_SW1	260	260	159	159	202	206	141	141	183	183	207	211
31Wr	G_SW1	260	270	159	165	202	206	141	143	183	189	203	215
9Wr	G_SW1	268	270	159	159	202	206	141	141	189	189	203	219
22Wr	G_SW1	260	270	159	159	202	202	141	143	183	189	215	219
2Wr	G_SW1	260	268	159	159	202	204	141	141	183	185	215	215
18Wr	G_SW1	260	270	159	171	200	200	141	141	183	189	215	219
19Wr	G_SW1	260	270	159	159	202	202	141	143	189	189	215	223
28Wr	G_SW1	268	270	159	159	206	206	141	143	183	183	203	211
7Wr	G_SW1	268	270	165	165	206	206	141	141	189	189	215	215

CODE	SITE	Mel1	Mel1	Mer041	Mer041	Ma1	Ma1	Mer43	Mer43	Mer15	Mer15	Mf4.17	Mf4.17
23Wr	G_SW1	260	268	159	159	202	206	141	141	183	189	215	223
27Wr	G_SW1	270	272	159	159	202	206	141	141	189	189	203	215
224km	G_SW1	260	270	159	159	202	202	141	141	183	185	211	219
346km	G_SW1	268	272	159	171	200	202	141	141	185	189	207	211
309km	H_W1	270	270	159	159	202	206	141	141	189	189	215	215
453km	H_W1	260	266	159	159	200	200	141	141	185	189	215	223
317km	H_W1	268	268	159	159	200	202	141	141	183	189	215	219
321km	H_W1	266	268	159	171	202	202	141	141	183	189	211	215
313km(488)	H_W1	260	270	159	159	202	206	141	141	183	183	211	215
320km	H_W1	268	270	159	171	202	206	141	141	183	183	215	223
491km	H_W1	270	270	159	159	202	204	141	141	183	183	211	215
322km(485)	H_W1	268	270	159	159	202	202	141	141	183	183	211	215
319km	H_W1	268	268	159	167	202	204	141	141	185	185	215	215
489km	H_W1	268	270	159	171	202	202	141	141	183	183	207	211
490km	H_W1	260	268	159	159	202	204	141	145	183	183	211	215
486km	H_W1	260	270	171	171	202	202	141	141	183	189	219	219

CODE	SITE	Mf8.8	Mf8.8	Mf8.10	Mf8.10	Mer08	Mer08	Mf1.3	Mf1.3
034km	A_NE1	231	243	120	140	149	149	194	194
111km	A_NE1	239	243	120	120	145	149	202	218
029F	A_NE1	231	239	120	140	145	149	202	202
154km	A_NE1	239	243	120	140	149	149	206	206
054F	A_NE1	239	239	120	140	149	149	194	202
022M	A_NE1	231	239	140	144	149	149	194	194
043F	A_NE1	231	239	140	144	149	149	194	194
004M	A_NE1	239	239	120	140	145	149	194	206
031M	A_NE1	231	239	120	144	149	149	202	202
025M(17M)	A_NE1	231	243	140	144	149	153	202	214
029M	A_NE1	247	247	132	144	145	149	202	206
369km	A_NE1	231	239	120	140	149	149	194	202
021F	A_NE1	231	231	120	140	149	153	202	202
052F(40F)	A_NE1	231	239	120	140	149	153	194	202
025F	A_NE1	239	247	140	144	145	149	194	206
024F(289)	A_NE1	231	239	140	144	149	149	202	214
035M	A_NE1	231	239	120	144	149	149	202	206
198km	A_NE1	239	243	140	144	145	149	194	202
026M	A_NE1	231	239	120	140	145	149	202	202
032M	A_NE1	231	239	120	140	145	153	202	214
110km	A_NE1	231	243	120	144	149	153	214	214
006_7M	A_NE1	231	239	120	140	149	149	214	214
002F	A_NE1	231	243	140	144	153	153	206	214
002km	A_NE1	231	243	120	120	145	149	202	218
008F	A_NE1	239	243	120	140	149	153	194	202
039F	A_NE1	231	247	120	120	149	149	194	202
197km	A_NE1	239	239	120	120	149	149	194	206
087km	A_NE1	239	239	120	140	145	149	194	194
009M	A_NE1	239	239	120	132	145	149	214	218
007M	A_NE1	239	243	120	140	145	149	214	218
001M	A_NE1	239	243	120	120	145	153	206	214
013km	A_NE1	231	239	120	140	149	149	206	206

CODE	SITE	Mf8.8	Mf8.8	Mf8.10	Mf8.10	Mer08	Mer08	Mf1.3	Mf1.3
031F	A_NE1	239	239	140	140	145	145	202	206
057km(5M)	A_NE1	239	243	120	140	145	153	202	214
047F	A_NE1	239	243	120	120	145	149	194	202
016M	A_NE1	239	239	120	120	145	149	194	218
106km	A_NE1	231	239	120	140	145	149	202	206
042F	A_NE1	231	243	120	140	145	145	206	214
023F	A_NE1	231	239	120	144	145	149	206	214
006M	A_NE1	231	239	120	140	145	149	202	218
050F	A_NE1	243	243	120	140	145	149	202	214
325km	A_NE1	239	247	120	120	149	149	206	206
38F	A_NE1	239	239	140	140	145	149	194	206
019M	A_NE1	231	239	120	140	145	153	194	214
049F	A_NE1	239	243	140	144	149	149	202	202
023M	A_NE1	231	239	120	140	149	149	206	214
090km	A_NE1	231	239	140	144	149	153	194	214
033M	A_NE1	239	243	140	140	145	145	194	214
028F	A_NE1	243	247	120	120	149	149	194	206
207km	A_NE1	231	231	140	140	145	149	202	218
055F	A_NE1	231	239	120	140	145	149	202	214
022km	A_NE1	231	243	120	120	149	149	202	202
056km	A_NE1	231	239	120	132	149	153	202	218
123km	A_NE1	239	239	120	132	145	149	206	218
113km	A_NE1	231	231	132	140	153	153	194	218
348km	A_NE1	239	243	120	140	145	149	206	206
112km	A_NE1	231	231	132	140	153	153	194	218
203km	A_NE1	239	239	120	120	145	149	194	218
450km	B_NE2	239	239	120	140	145	153	194	206
499km	B_NE2	231	243	120	120	145	149	202	202
296km	B_NE2	231	239	120	140	149	149	194	194
349km	B_NE2	231	243	120	132	145	153	202	206
354km	B_NE2	243	243	120	120	149	149	194	214
225km	B_NE2	239	239	132	140	149	149	214	218

CODE	SITE	Mf8.8	Mf8.8	Mf8.10	Mf8.10	Mer08	Mer08	Mf1.3	Mf1.3
316km	B_NE2	231	243	120	140	145	149	206	222
396km	B_NE2	231	231	120	140	145	149	194	218
315km	B_NE2	247	247	120	140	149	149	194	202
223km	B_NE2	231	247	120	140	149	149	194	214
045F	B_NE2	231	239	140	140	145	153	202	218
003km	B_NE2	231	243	120	120	149	149	202	214
393km	B_NE2	243	247	132	140	153	153	194	218
018M	B_NE2	239	247	120	132	149	153	218	218
046F	B_NE2	231	239	120	140	149	149	194	214
20F	B_NE2	243	247	120	132	149	153	218	218
028M	B_NE2	231	247	120	140	145	149	206	218
034M	B_NE2	239	239	140	140	153	153	218	218
226km	B_NE2	231	231	120	120	149	149	194	206
022F	B_NE2	243	243	140	140	149	149	194	194
037F	B_NE2	239	239	120	120	153	153	194	202
053F	B_NE2	239	239	120	120	149	153	194	194
020M	B_NE2	239	243	120	120	153	153	194	194
493km	B_NE2	243	247	120	120	145	149	194	194
536km	B_NE2	247	247	140	140	149	149	194	218
021M(451)	B_NE2	231	239	140	140	149	153	194	218
051F	B_NE2	231	231	140	140	149	153	194	218
044F	B_NE2	231	239	120	140	149	153	194	194
027M	B_NE2	231	231	140	140	153	153	194	194
239km	B_NE2	239	247	132	140	145	149	194	218
368km	B_NE2	239	247	120	120	145	153	194	206
048F	B_NE2	231	247	140	140	153	153	194	218
147km	B_NE2	239	247	120	140	149	153	194	194
034F	B_NE2	231	231	140	140	149	153	194	218
116km	B_NE2	231	243	120	120	147	149	194	194
210km	B_NE2	247	251	120	132	145	149	194	218
405km	B_NE2	243	247	140	140	149	149	206	206
323km	B_NE2	239	247	132	140	149	149	202	206

CODE	SITE	Mf8.8	Mf8.8	Mf8.10	Mf8.10	Mer08	Mer08	Mf1.3	Mf1.3
476km	B_NE2	231	243	132	140	149	149	194	218
297km	B_NE2	231	231	120	132	149	149	194	194
324km	B_NE2	231	243	140	140	145	149	194	194
241km	B_NE2	231	243	120	132	145	149	194	206
217km	B_NE2	231	247	120	140	145	153	194	218
138km	B_NE2	231	243	120	140	149	149	194	206
455km	B_NE2	231	243	0	0	153	153	202	202
211km	B_NE2	231	231	120	140	149	153	194	194
222km	B_NE2	239	247	140	140	149	149	194	194
108km	B_NE2	231	247	132	140	149	153	194	202
085km	B_NE2	243	247	120	140	145	149	194	206
127km	B_NE2	239	247	120	140	149	149	206	214
044km	B_NE2	239	239	120	120	149	149	202	206
282km	B_NE2	231	243	120	132	149	153	194	214
403km	B_NE2	239	251	120	120	145	153	194	218
365km	B_NE2	239	239	132	140	149	149	194	194
371km	B_NE2	247	247	132	140	145	149	194	214
137km	B_NE2	239	239	120	120	145	149	194	206
299km	B_NE2	243	247	120	120	149	149	214	218
362-2km	B_NE2	231	247	120	140	149	153	194	206
308km	B_NE2	247	247	120	120	153	153	194	194
140km	B_NE2	231	247	140	140	149	149	194	206
238km	B_NE2	239	239	120	140	145	153	202	206
402km	B_NE2	239	239	140	140	145	153	194	194
397km	B_NE2	239	243	140	140	149	153	194	194
270km	B_NE2	231	239	132	140	145	153	206	206
276km	B_NE2	231	239	120	140	149	153	194	202
474km	B_NE2	231	239	120	120	149	149	202	202
220_7km	B_NE2	239	251	120	132	145	149	202	206
220_6km	B_NE2	239	247	140	140	143	147	202	218
220_2km	B_NE2	231	239	140	140	145	149	206	218
220_4km	B_NE2	239	251	120	140	149	149	202	206

CODE	SITE	Mf8.8	Mf8.8	Mf8.10	Mf8.10	Mer08	Mer08	Mf1.3	Mf1.3
220_3km	B_NE2	243	243	140	140	153	153	194	206
220_1km	B_NE2	239	239	120	140	145	149	202	206
267km	B_NE2	239	239	132	132	139	139	206	218
372km	B_NE2	231	243	120	120	149	153	194	218
287km	B_NE2	247	255	120	120	149	149	194	214
286km	B_NE2	231	239	120	140	145	149	194	194
284km	B_NE2	239	243	120	120	149	153	194	194
283km	B_NE2	231	231	120	120	145	149	194	194
281km	B_NE2	231	239	120	140	145	145	194	194
292km	B_NE2	231	239	120	120	149	153	194	218
295km	B_NE2	231	243	120	140	145	149	194	214
234km	B_NE2	231	239	140	140	149	149	194	214
377km	B_NE2	231	247	120	120	145	145	194	214
437km	B_NE2	231	239	140	140	145	149	206	214
229km	B_NE2	231	231	120	140	145	145	194	206
269km	B_NE2	231	231	0	0	149	153	194	218
357km	B_NE2	231	247	132	140	145	149	194	218
358km	B_NE2	231	239	120	132	145	149	214	214
237km	B_NE2	231	243	120	132	149	149	194	194
376km	B_NE2	239	239	132	140	145	145	194	202
503km	B_NE2	243	251	120	140	149	153	194	194
367km	B_NE2	0	0	120	140	145	149	194	202
404km	B_NE2	239	247	120	132	149	153	194	194
406km	B_NE2	231	239	132	132	145	149	202	214
208km	B_NE2	243	247	120	120	145	153	194	194
522km	C_NE3	243	247	140	140	149	153	194	194
446km	C_NE3	231	231	132	140	153	153	194	218
543km	C_NE3	239	243	140	140	153	153	194	194
353_1km(PC	C_NE3	219	235	136	140	147	149	202	214
527km	C_NE3	239	247	140	140	145	153	206	218
448km	C_NE3	231	243	140	140	149	149	194	206
274km	C_NE3	239	239	120	120	145	145	206	218

CODE	SITE	Mf8.8	Mf8.8	Mf8.10	Mf8.10	Mer08	Mer08	Mf1.3	Mf1.3
311km	C_NE3	239	239	120	120	145	149	194	194
248km	C_NE3	231	243	120	120	149	149	194	194
244km	C_NE3	239	239	140	140	149	153	194	218
443km	C_NE3	247	247	132	140	145	153	194	214
246km	C_NE3	231	247	120	120	153	153	194	202
440km	C_NE3	239	247	120	132	153	153	194	206
442km	C_NE3	239	243	140	140	145	149	202	214
441km	C_NE3	247	247	120	140	149	153	194	202
179km	C_NE3	231	247	120	140	149	153	194	214
245-akm	C_NE3	231	231	120	140	149	153	194	194
245km	C_NE3	239	247	120	140	153	153	194	206
232_1km	C_NE3	231	239	120	140	151	153	214	218
232_2km	C_NE3	239	247	120	120	149	149	194	202
436km	C_NE3	231	239	120	140	149	153	194	194
249km	C_NE3	235	243	140	144	153	153	194	202
243km	C_NE3	239	247	120	140	149	149	194	218
247km	C_NE3	239	243	140	140	153	153	194	202
449km	C_NE3	231	247	120	132	149	153	194	194
086km	D_CE1	247	251	120	120	145	145	194	206
391km	D_CE1	239	239	120	140	149	153	194	194
169km	D_CE1	219	247	132	140	149	149	194	214
326km	D_CE1	231	251	120	132	145	149	194	214
300km	D_CE1	231	247	120	144	145	145	194	202
411km	D_CE1	239	239	140	140	153	153	194	194
115km(PCR1	D_CE1	231	239	120	140	153	153	194	202
395km	D_CE1	231	239	120	140	149	153	194	214
305km	D_CE1	231	239	140	148	149	149	194	194
167km	D_CE1	231	231	120	140	145	149	194	202
519km	D_CE1	231	247	120	132	149	153	194	218
475km	D_CE1	231	243	140	144	149	149	194	202
242km	D_CE1	243	247	120	140	145	149	194	206
228km	D_CE1	231	251	120	140	149	153	194	214

CODE	SITE	Mf8.8	Mf8.8	Mf8.10	Mf8.10	Mer08	Mer08	Mf1.3	Mf1.3
212km	D_CE1	231	247	120	140	149	149	202	218
107km	E_CE2	219	247	120	132	145	149	194	202
484km	E_CE2	231	247	148	148	153	153	194	214
480km	E_CE2	231	243	120	120	149	153	194	206
481km	E_CE2	243	251	136	140	145	145	194	214
398km	E_CE2	231	231	132	140	149	149	202	206
252km	E_CE2	239	239	136	140	145	149	194	194
253km	E_CE2	243	247	120	144	145	153	194	194
236km	E_CE2	239	247	120	132	149	149	194	202
260km	E_CE2	231	247	120	132	145	149	202	206
250km	E_CE2	239	247	120	136	149	153	194	194
262km	E_CE2	219	235	120	132	149	153	194	202
265km	E_CE2	231	247	120	140	149	153	194	206
168km	E_CE2	239	247	120	140	149	149	194	202
258km	E_CE2	231	243	132	136	149	153	202	202
255km	E_CE2	243	247	120	136	153	153	206	218
256km	E_CE2	235	247	120	120	149	149	194	194
264km	E_CE2	231	239	120	132	149	149	194	214
251km	E_CE2	235	239	140	140	149	149	194	206
261km	E_CE2	219	247	120	120	145	149	194	194
257km	E_CE2	231	239	132	140	149	153	206	218
254km	E_CE2	231	243	120	132	149	153	194	194
263km	E_CE2	219	231	132	140	149	149	194	194
333km	F_SE1	239	243	140	144	145	149	214	214
29Wr	F_SE1	239	251	120	132	145	151	214	214
386km	F_SE1	239	251	140	140	149	149	214	214
388km	F_SE1	231	247	140	144	145	151	194	202
383km	F_SE1	231	231	120	140	145	149	202	202
381km	F_SE1	247	251	132	144	145	149	194	194
385km	F_SE1	239	247	132	132	145	153	194	202
382km	F_SE1	243	243	120	140	149	149	202	206
380km	F_SE1	239	239	120	140	149	153	194	194

CODE	SITE	Mf8.8	Mf8.8	Mf8.10	Mf8.10	Mer08	Mer08	Mf1.3	Mf1.3
384km	F_SE1	231	243	140	140	149	149	194	194
390km	F_SE1	231	231	132	140	149	151	194	202
387km	F_SE1	231	247	132	144	145	145	194	194
389km	F_SE1	235	247	120	140	145	149	194	194
378km	F_SE1	239	243	120	132	151	153	202	202
1Wr	G_SW1	231	231	120	120	153	153	202	214
16Wr	G_SW1	231	231	132	148	145	153	202	206
11Wr	G_SW1	231	231	140	144	145	149	202	214
6Wr	G_SW1	231	247	144	144	145	145	206	214
15Wr	G_SW1	231	231	120	140	145	153	194	194
13Wr	G_SW1	219	231	140	148	153	153	194	206
10Wr	G_SW1	231	247	120	140	153	153	194	194
12Wr	G_SW1	231	239	132	140	145	145	206	206
17Wr	G_SW1	231	247	144	148	145	153	194	194
26Wr	G_SW1	231	243	120	140	153	153	194	194
21Wr	G_SW1	235	243	132	132	145	153	206	206
8Wr	G_SW1	243	247	120	132	149	153	206	206
4Wr	G_SW1	231	243	140	140	153	153	202	206
25Wr	G_SW1	231	231	120	148	145	145	194	202
30Wr	G_SW1	231	247	132	148	153	153	194	194
3Wr	G_SW1	231	243	132	140	149	153	202	214
14Wr	G_SW1	247	247	132	144	153	153	202	214
20Wr	G_SW1	243	243	132	140	153	153	206	218
5Wr	G_SW1	231	239	140	144	145	145	194	202
31Wr	G_SW1	227	247	140	144	147	149	202	214
9Wr	G_SW1	231	247	120	136	145	153	194	206
22Wr	G_SW1	235	239	132	144	149	149	194	214
2Wr	G_SW1	239	243	120	140	153	153	202	214
18Wr	G_SW1	235	239	132	144	153	153	194	194
19Wr	G_SW1	231	239	132	132	153	153	206	214
28Wr	G_SW1	231	239	132	140	145	153	194	202
7Wr	G_SW1	239	243	136	136	149	153	194	202

CODE	SITE	Mf8.8	Mf8.8	Mf8.10	Mf8.10	Mer08	Mer08	Mf1.3	Mf1.3
23Wr	G_SW1	239	247	132	140	149	153	194	202
27Wr	G_SW1	235	239	120	140	145	153	194	202
224km	G_SW1	235	239	132	136	149	153	194	194
346km	G_SW1	231	247	120	120	153	153	194	202
309km	H_W1	231	235	140	140	153	153	194	206
453km	H_W1	231	239	136	140	147	153	194	202
317km	H_W1	247	247	132	136	149	153	194	206
321km	H_W1	231	239	120	140	145	153	194	194
313km(488)	H_W1	235	239	132	140	153	153	206	214
320km	H_W1	231	235	132	140	151	153	194	194
491km	H_W1	239	247	120	132	153	153	194	202
322km(485)	H_W1	231	247	132	132	149	153	194	202
319km	H_W1	239	239	120	132	153	153	194	206
489km	H_W1	247	247	140	140	149	153	194	206
490km	H_W1	247	247	140	140	145	153	194	214
486km	H_W1	231	247	132	132	147	149	194	214
